# Identification and characterization of *Sr22b*, a new allele of the wheat stem rust resistance gene *Sr22* effective against the Ug99 race group

**DOI:** 10.1111/pbi.13737

**Published:** 2021-11-06

**Authors:** Jing Luo, Matthew N. Rouse, Lei Hua, Hongna Li, Boshu Li, Tianya Li, Wenjun Zhang, Caixia Gao, Yanpeng Wang, Jorge Dubcovsky, Shisheng Chen

**Affiliations:** ^1^ Peking University Institute of Advanced Agricultural Sciences Weifang Shandong 261000 China; ^2^ USDA‐ARS Cereal Disease Laboratory and Department of Plant Pathology University of Minnesota St. Paul MN 55108 USA; ^3^ State Key Laboratory of Plant Cell and Chromosome Engineering Center for Genome Editing Institute of Genetics and Developmental Biology The Innovative Academy of Seed Design Chinese Academy of Sciences Beijing China; ^4^ College of Plant Protection Shenyang Agricultural University Shenyang Liaoning 110000 China; ^5^ Department of Plant Sciences University of California Davis CA 95616 USA; ^6^ Howard Hughes Medical Institute Chevy Chase MD 20815 USA

**Keywords:** *Sr22b*, stem rust, resistance gene, CC‐NBS‐LRR, introgression, wheat

## Abstract

Wheat stem (or black) rust, caused by *Puccinia graminis* f. sp. *tritici* (*Pgt*), has been historically among the most devastating global fungal diseases of wheat. The recent occurrence and spread of new virulent races such as Ug99 have prompted global efforts to identify and isolate more effective stem rust resistance (*Sr*) genes. Here, we report the map‐based cloning of the Ug99‐effective *SrTm5* gene from diploid wheat *Triticum monococcum* accession PI 306540 that encodes a typical coiled‐coil nucleotide‐binding leucine‐rich repeat protein. This gene, designated as *Sr22b*, is a new allele of *Sr22* with a rare insertion of a large (13.8‐kb) retrotransposon into its second intron. Biolistic transformation of an ~112‐kb circular bacterial artificial chromosome plasmid carrying *Sr22b* into the susceptible wheat variety Fielder was sufficient to confer resistance to stem rust. In a survey of 168 wheat genotypes, *Sr22b* was present only in cultivated *T*. *monococcum* subsp. *monococcum* accessions but absent in all tested tetraploid and hexaploid wheat lines. We developed a diagnostic molecular marker for *Sr22b* and successfully introgressed a *T*. *monococcum* chromosome segment containing this gene into hexaploid wheat to accelerate its deployment and pyramiding with other *Sr* genes in wheat breeding programmes. *Sr22b* can be a valuable component of gene pyramids or transgenic cassettes combining different resistance genes to control this devastating disease.

## Introduction

Wheat is an important cereal crop that contributes a substantial proportion of the calories and proteins consumed by humankind. Reducing yield losses inflicted by pathogens can contribute to grain yield improvements that are required to feed a growing world population. *Puccinia graminis* f. sp. *tritici* (*Pgt*), the causal agent of wheat stem rust (or black rust), has historically been a devastating fungal disease of tetraploid and hexaploid wheat. In the past, this pathogen was effectively controlled by growing resistant wheat varieties and eradicating alternate host (*Berberis vulgaris*) plants around cereal fields (Roelfs, 1982, 1985; Singh *et al*., 2015).

After the year 1998, this disease became a major concern again after the emergence and spread of the *Pgt* race TTKSK (Ug99) and its variants (henceforth the Ug99 race group), which were virulent on the majority of resistance genes deployed worldwide, including resistance genes *Sr31* and *Sr38* (Pretorius *et al*., 2000; Singh *et al*., 2006, 2011). In recent years, additional highly virulent *Pgt* races unrelated to Ug99, such as TRTTF, TKTTF and TTRTF (Olivera *et al*., 2012, 2015; Patpour *et al*., 2017; Tesfaye *et al*., 2020), have been detected in outbreaks in Africa (Olivera *et al*., 2015), Asia (Shamanin *et al*., 2016, 2018) and Europe (Bhattacharya, 2017; Olivera *et al*., 2017). Due to the threat of these new virulent *Pgt* races, there is an urgent need to identify and isolate new effective *Sr* genes to diversify the sources of resistance in wheat breeding programmes.

Over 60 stem rust resistance genes (*Sr1–Sr61*) have been assigned official designations (Chen *et al*., 2020; Zhang *et al*., 2020), among which a large proportion were introgressed from wild wheat relatives (Singh *et al*., 2015). The diploid wheat species *Triticum monococcum* (einkorn, genome A^m^), comprising of the domesticated *T*. *monococcum* ssp. *monococcum* and the wild *T*. *monococcum* ssp. *aegilopoides*, is closely related to *T. urartu* (genome A^u^), the donor of the A genome in polyploid wheat (Dvorak and McGuire, 1988). *T*. *monococcum* harbours several valuable rust resistance genes, including the leaf rust resistance genes *LrTM16* (Sodkiewicz and Strzembicka, 2008) and *Lr63* (Kolmer and Anderson, 2010); the stripe rust resistance loci *QYrtm.pau‐2A* and *QYrtb.pau‐5A* (Chhuneja *et al*., 2008) and *Yr34* (Chen *et al*., 2021); and the stem rust resistance genes *Sr21* (Chen *et al*., 2015; The, 1973), *Sr22* (Gerechter‐Amitai *et al*., 1971), *Sr35* (McIntosh *et al*., 1984), *SrTm4* (Briggs *et al*., 2015) and *Sr60* and *SrTm5* (Chen *et al*., 2018a).

*Triticum monococcum* chromosomes can recombine with the A‐genome chromosomes of polyploid wheat, particularly in the presence of the *ph1b* mutation (Dubcovsky and Luo, 1995). This feature has fuelled interest of scientists and breeders in the identification and isolation of stem rust resistance genes from this species and its transfer to commercial wheat cultivars. Among the six stem rust resistance genes derived from *T*. *monococcum*, four officially named ones (*Sr21*, *Sr22*, *Sr35* and *Sr60*) have been successfully cloned and transferred into hexaploid wheat so far (Chen *et al*., 2020; Chen *et al*., 2018b; Saintenac *et al*., 2013; Steuernagel *et al*., 2016). The first three are Ug99‐resistance genes encoding typical coiled‐coil nucleotide‐binding leucine‐rich repeat (CC‐NBS‐LRR) proteins (Chen *et al*., 2018b; Saintenac *et al*., 2013; Steuernagel *et al*., 2016), whereas *Sr60* encodes a different type of protein with two putative kinase domains (Chen *et al*., 2020).

Cultivated *T*. *monococcum* accession PI 306540 was identified as having a unique resistance response to five *Pgt* isolates (Rouse and Jin, 2011a, b), which was subsequently associated with the presence of stem rust resistance genes *SrTm4*, *Sr21*, *Sr60* and *SrTm5* (Briggs *et al*., 2015; Chen *et al*., 2018a, b). *SrTm5* was previously mapped to the same region as *Sr22* on the long arm of chromosome 7A^m^, and showed good levels of resistance (IT = ; to ;1) to several *Pgt* races, including TTKSK, TTKST and MCCFC (Chen *et al*., 2018a). Based on its mapped location and its different resistance profiles from *Sr22*, it was hypothesized that *SrTm5* could be a novel allele of *Sr22* or a tightly linked gene (Chen *et al*., 2018a).

In this study, we describe the map‐based cloning of the stem rust resistance gene *SrTm5*, and confirm that it is a new allele of the cloned gene *Sr22*. SrTm5 was roughly 96% identical to the reported Sr22 proteins and showed a characteristic insertion of 13.8‐kb retrotransposon in its second intron. We successfully introgressed a *T*. *monococcum* chromosome segment carrying *SrTm5* into hexaploid wheat and developed a diagnostic molecular marker to accelerate its deployment in wheat breeding programmes.

## Results

### Assessment of stem rust responses

At the seedling stage, the *SrTm5* monogenic line TmR54‐3 exhibited high levels of resistance (Its = ; to ;1) to *Pgt* races 34PKUSC, 34MTGSM and TTKSK, but was susceptible (ITs = 3+) to the other three races BCCBC, 21C3CTTTM and RTJRM. By contrast, its sister line TmS57‐57 without *SrTm5* displayed susceptible infection types (ITs = 3+) to all the tested races (Figure S1a and Table S1). When inoculated with race 34PKUSC, selected F_5_ families from the *SrTm5* segregating mapping population showed infection types that ranged from ‘;’ to ‘1’ in resistant plants, and from ‘3’ to ‘4’ in susceptible plants (Figure S1b).

To quantify the infected leaf area, we measured the percentage of the leaf area covered with *Pgt* pustules on six independent infected leaves of TmR54‐3 and TmS57‐57 using the software ASSESS version 2. For *SrTm5*‐avirulent races 34PKUSC, 34MTGSM and TTKSK, the average percentage was significantly lower (*P* < 0.001) in plants carrying *SrTm5* than in those without the gene (Figure S1).

### Map‐based cloning of *SrTm5*

**Figure 1:**
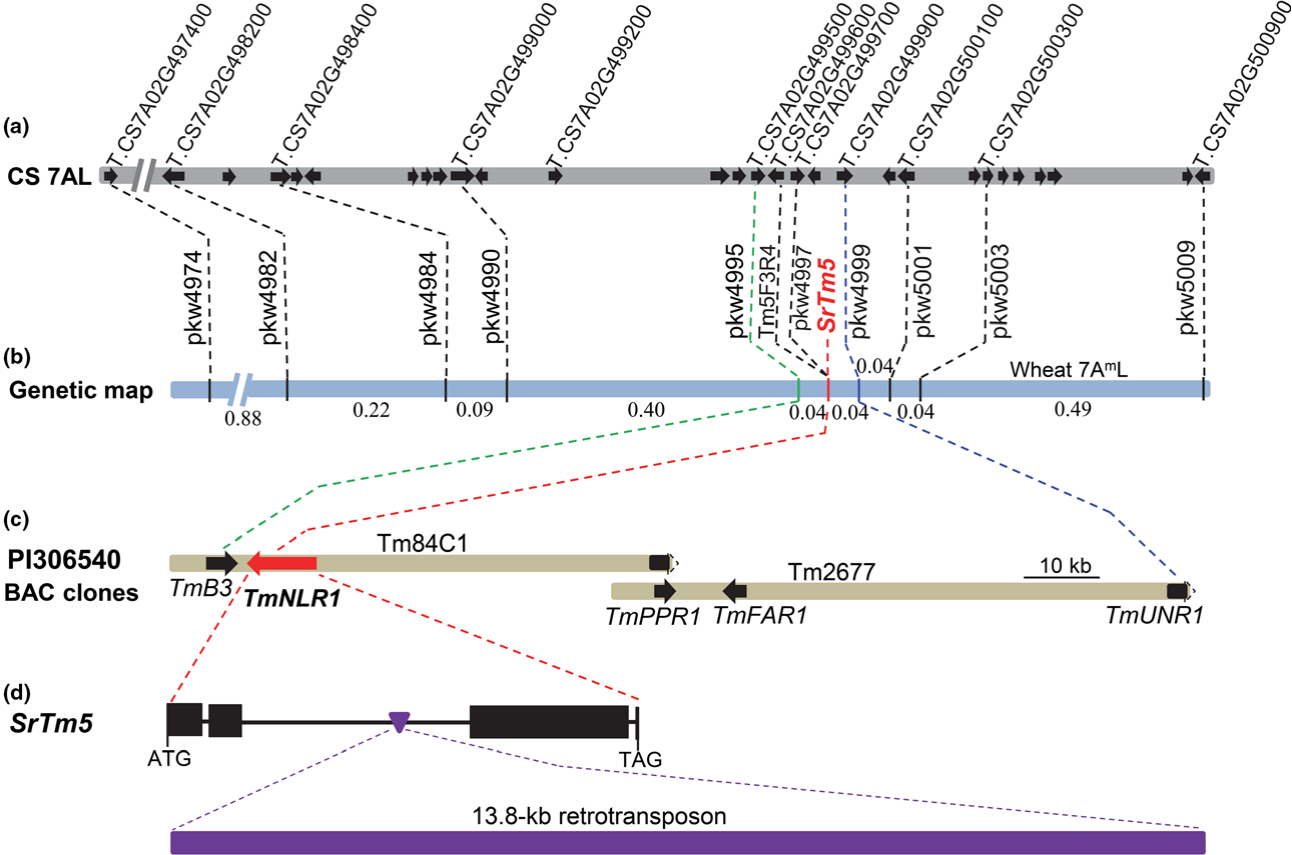
Map‐based cloning of *SrTm5*. (a) Collinear region on chromosome arm 7AL of Chinese Spring (RefSeq v1.1). Arrows represent genes. (b) High‐density genetic map of *SrTm5* using 2264 segregating gametes. (c) Predicted genes in the *SrTm5* candidate region constructed with two overlapping BACs from the resistant parent PI 306540. Dotted lines in arrows indicate deleted partial gene coding regions in BACs. (d) Gene structure of *SrTm5* in PI 306540. Black rectangles indicate exons and black lines indicate introns; the purple inverted triangle in the second intron indicates the insertion of a retrotransposon.

The initial mapping of *SrTm5* suggested that this gene was either a novel allele of *Sr22* (*TraesCS7A02G499600*) or a tightly linked gene (Chen *et al*., 2018a). Since *Sr22* is located on the long arm of chromosome 7A at 689.9 Mb (Chinese Spring RefSeq v1.0; The International Wheat Genome Sequencing Consortium, 2018), we developed Cleaved Amplified Polymorphic Sequence (CAPS) markers *pkw4974* (690.9 Mb) and *pkw5009* (688.2 Mb) (Table 1) flanking the *Sr22* locus. Subsequently, we used these two markers to screen a population of 1132 plants (2264 gametes) from the cross PI 306540 × PI 272557, and we found 51 plants carrying recombination events within this region (2.7 Mb or 2.3 cM). Evaluations of progeny of these plants with race 34PKUSC confirmed that *SrTm5* was located within this region. Using nine new markers spanning the 2.7 Mb (Table 1), we further delimited the *SrTm5* candidate region to a 0.08‐cM interval (140.4 kb, CS RefSeq v1.0 coordinates) flanked by CAPS markers *pkw4995* and *pkw4999* (Figure 1b).

**Table 1:** Primers used in the present study

Marker	ID in CS RefSeq v1.1	Primer sequence 5'–3' (Forward)	Primer sequence 5'–3' (Reverse)	Size (bp)	Enzyme	Function
*pkw4974*	*TraesCS7A02G497400*	GCACTCCAGGTGTCGCTCAG	ACCATTTCTCGCCGCTGTTC	619	*Hae*III	Fine mapping
*pkw4982*	*TraesCS7A02G498200*	GTATGTGAAATAGAAAATGGGCAAC	CATAAGATTGCTGCCAAAGAACT	944	*Mfe*I	Fine mapping
*pkw4984*	*TraesCS7A02G498400*	CCATTTGCTCCCACGAACA	CCCCATCAAGCCACTCTAT	607	*Mbo*II	Fine mapping
*Pkw4990*	*TraesCS7A02G499000*	TGAAAGGGAAGGTGAAGGA	AGGTGGAGGTTAAGGCGAG	970	*BsaJ*I	Fine mapping
*pkw4995*	*TraesCS7A02G499500*	CTCAGAACACGGCTTCAACA	GATCACATGGACCTTCATCG	900	*Ssp*I	Fine mapping
*Tm5F3R4*	*TraesCS7A02G499600*	TGGAGAAAGTGGACAAGAT	GCTGCTCTATCTTCGGTTG	971	*Pvu*II	Fine mapping
*TM5TF3R3*	*TraesCS7A02G499600*	GGATTTAGGGTTTCGGGGA	CCAACTACCACCACGGACG	1137	–	Fine mapping
*pkw4997*	*TraesCS7A02G499700*	TATGCCCAAAAGGAGTAGG	TACATCCTGTAGGACAAAACTG	709	*Acc*I	Fine mapping
*pkw4999*	*TraesCS7A02G499900*	TGTCTACTGCATGAAGTTCAACC	AGCGGTCTCATTGACGGAA	799	*Aat*II	Fine mapping
*pkw5001*	*TraesCS7A02G500100*	CGGTGTAGCATACCATTTCG	TTTCTTGTAGAGCGGGAGC	1448	–	Fine mapping
*pkw5003*	*TraesCS7A02G500300*	CTGTTGCTCAACGCCCATCTC	GATCACGTCGGGCATGAACTTATA	675	*Sma*I	Fine mapping
*pkw5009*	*TraesCS7A02G500900*	TCTTGCTGTTGCTTGGCTGTC	TGTCCCGCCTGTTGTTCCT	1205	*Sph*I	Fine mapping
*TM5TF2R2*	*TraesCS7A02G499600*	GCACTGAGACTCCTCGGTGATGT	CACTCATATTACCCCCTTCCTTACC	673	–	MAS
*A120F6R6*	*TraesCS7A02G499600*	AAGAACTTGCTGCCGGACAT	AATCTTGTACCTTGAAAATCTGTCG	108	–	Expression analysis
*HL‐F61R60*	*TraesCS7A02G499600*	GTTGCAGAGTTTTCGGGTTTACC	GGCTTTCCGATGAAGTCATAGAA	109	–	Expression analysis
*4997QF2R2*	*TraesCS7A02G499700*	CCAAAAGGAGTAGGAGTACA	ACGCATCATATCAAAGAAAC	260	–	Semi‐quantitative PCR
*4998QF5R5*	*TraesCS7A02G499800*	CATTCTAAAGGTGTGATGGATTA	ATTGGCCTTTCTGAGGTTGG	272	‐	Semi‐quantitative PCR
*TM5AF6R8*	*TraesCS7A02G499600*	CTAGACAATTACATCAAGGTATA	GGGTATCAATCCAATCATCTCAATA	1688		Sequencing
*TM5AF4R4*	*TraesCS7A02G499600*	GGTGTCCTCTCTCTGTAAACTGG	ATCTATTTGCTCGTCTCGTAACATA	649		Sequencing
*cfa2049*	–	TAATTTGATTGGGTCGGAGC	CGTGTCGATGGTCTCCTTG		–	Introgression
*wmc405*	–	GTGCGGAAAGAGACGAGGTT	TATGTCCACGTTGGCAGAGG		–	Introgression
*cfd68*	–	TTTGCAGCATCACACGTTTT	AAAATTGTATCCCCCGTGGT		–	Introgression
*gwm260*	–	GCCCCCTTGCACAAATC	CGCAGCTACAGGAGGCC		–	Introgression
*barc108*	–	GCGGGTCGTTTCCTGGAAATTCATCTAA	GCGAAATGATTGGCGTTACACCTGTTG		–	Introgression
*barc121*	–	ACTGATCAGCAATGTCAACTGAA	CCGGTGTCTTTCCTAACGCTATG		–	Introgression
*wmc790*	–	AATTAAGATAGACCGTCCATATCATCCA	CGACAACGTACGCGCC		–	Introgression

Only three complete genes (*TraesCS7A02G499600*, *TraesCS7A02G499700* and *TraesCS7A02G499800*) were annotated in the Chinese Spring reference genome within this region (Figure 1a). To determine if additional genes were present in the orthologous region in *T*. *monococcum*, we screened the bacterial artificial chromosome (BAC) library of resistant parent PI 306540 using the two flanking markers (*pkw4995* and *pkw4999*) and two markers completely linked to *SrTm5* (*Tm5F3R4* and *pkw4997*). We obtained two overlapping BAC clones designated hereafter as Tm84C1 and Tm2677. Sequencing and annotation of these two selected BACs (Figure 1c; GenBank accession MZ327628) showed no additional genes in the *SrTm5* candidate region in PI 306540 (146.5 kb) relative to Chinese Spring.

We designated the *T*. *monococcum* orthologues of Chinese Spring genes *TraesCS7A02G499600*, *TraesCS7A02G499700* and *TraesCS7A02G499800* as *TmNLR1*, *TmPPR1* and *TmFAR1* respectively. *TmPPR1* encodes a protein containing pentatricopeptide repeat domains, whereas *TmFAR1* encodes a far1‐related sequence 5‐like protein. We were not able to detect transcripts of these two genes in the leaves of *SrTm5*‐resistant *T*. *monococcum* plants infected with *Pgt* (Figure S2), suggesting that they are unlikely candidate genes for *SrTm5*.

*TmNLR1* is an orthologue of the cloned stem rust resistance gene *Sr22* (*TraesCS7A02G499600*) (Steuernagel *et al*., 2016) and therefore an excellent candidate gene for *SrTm5*. In PI 306540, the *TmNLR1* gene spans 19715 bp from start to stop codons, including the insertion of a 13.8‐kb gypsy‐like retrotransposon in the second intron (Figure 1d). Comparing the *TmNLR1* genomic region with the full‐length complementary DNA (cDNA) of *TmNLR1*, we determined that this gene contains four exons. The 2817 bp coding sequence encodes a typical CC‐NBS‐LRR protein containing 938 amino acids that were 95.7%–96.7% identical to six reported Sr22‐resistant protein haplotypes (Figure S3).

Three lines of evidence support *TmNLR1* as the best candidate for *SrTm5*. First, *TmNLR1* is the only candidate gene that is expressed in infected leaves of the resistant parent. Second, the TmNLR1 allele from PI 306540 shares the diagnostic amino acids present in known Sr22‐resistant alleles, whereas PI 272557 shares the diagnostic amino acids for the susceptible alleles (V381L, S605F/Y and G655D, BLOSUM62 scores = 1, −2 and −1, Table S2). Finally, sequencing of *TmNLR1* in *T*. *monococcum* accession PI 277131‐2, which was previously postulated to possess *SrTm5* (Rouse and Jin, 2011a), confirmed the presence of a gene 100% identical to *TmNLR1*. Based on these results, we selected *TmNLR1* for further functional characterizations.

### Validation of *TmNLR1* by transgenic complementation

To test if *TmNLR1* was sufficient to confer resistance to *Pgt*, we transformed the Ug99‐susceptible wheat variety Fielder with the PI 306540 circular BAC plasmid Tm84C1, which includes two complete genes *TmB3* and *TmNLR1*, and about 70% of the coding sequence of *TmPPR1* (Figure 1c). Gene *TmB3* is orthologous to Chinese Spring gene *TraesCS7A02G499500* and encodes a B3 domain‐containing protein likely to be involved in plant growth and development (Peng and Weselake, 2013; Waltner *et al*., 2005). Among them, only *TmNLR1* was expressed in infected leaves and co‐segregated with the disease phenotypes.

We obtained eight independent T_0_ transgenic plants, for which we confirmed the presence of the *TmNLR1* transgene using markers *Tm5F3R4*, *TM5TF2R2* and *TM5TF3R3* (Table 1). We genotyped more than 20 T_1_ plants from each transgenic family, and all except one showed significant segregation distortion from the 3 : 1 (transgenic/non‐transgenic) segregation expected from a single copy of transgene, with an excess of non‐transgenic plants (Table S3). We also genotyped T_2_ plants derived from one single positive T_1_ plant per event. Families T_2_‐Tm505‐15, T_2_‐Tm514‐2, T_2_‐Tm517‐1, T_2_‐Tm548‐3, T_2_‐Tm554‐2 and T_2_‐Tm558‐7 were fixed for the transgene (all plants are positive). Families T_2_‐Tm515‐6 and T_2_‐Tm547‐3 displayed a distorted segregation ratio from the expected 3+ : 1‐ with an excess of non‐transgenic plants close to a 1 : 1 segregation (Table S3). Taken together, these results suggest some segregation distortion against the transgene.

Transcript levels of *TmNLR1* in all transgenic T_1_ families were significantly higher than in the susceptible control Fielder (*P* < 0.01), but only five of them (T_1_‐Tm514, T_1_‐Tm515, T_1_‐Tm517, T_1_‐Tm548 and T_1_‐Tm554) were expressed at similar levels as in the introgression of the *T*. *monococcum* chromosome segment including *SrTm5* into Fielder (positive control, see later) (Figure S4).

**Figure 2:**
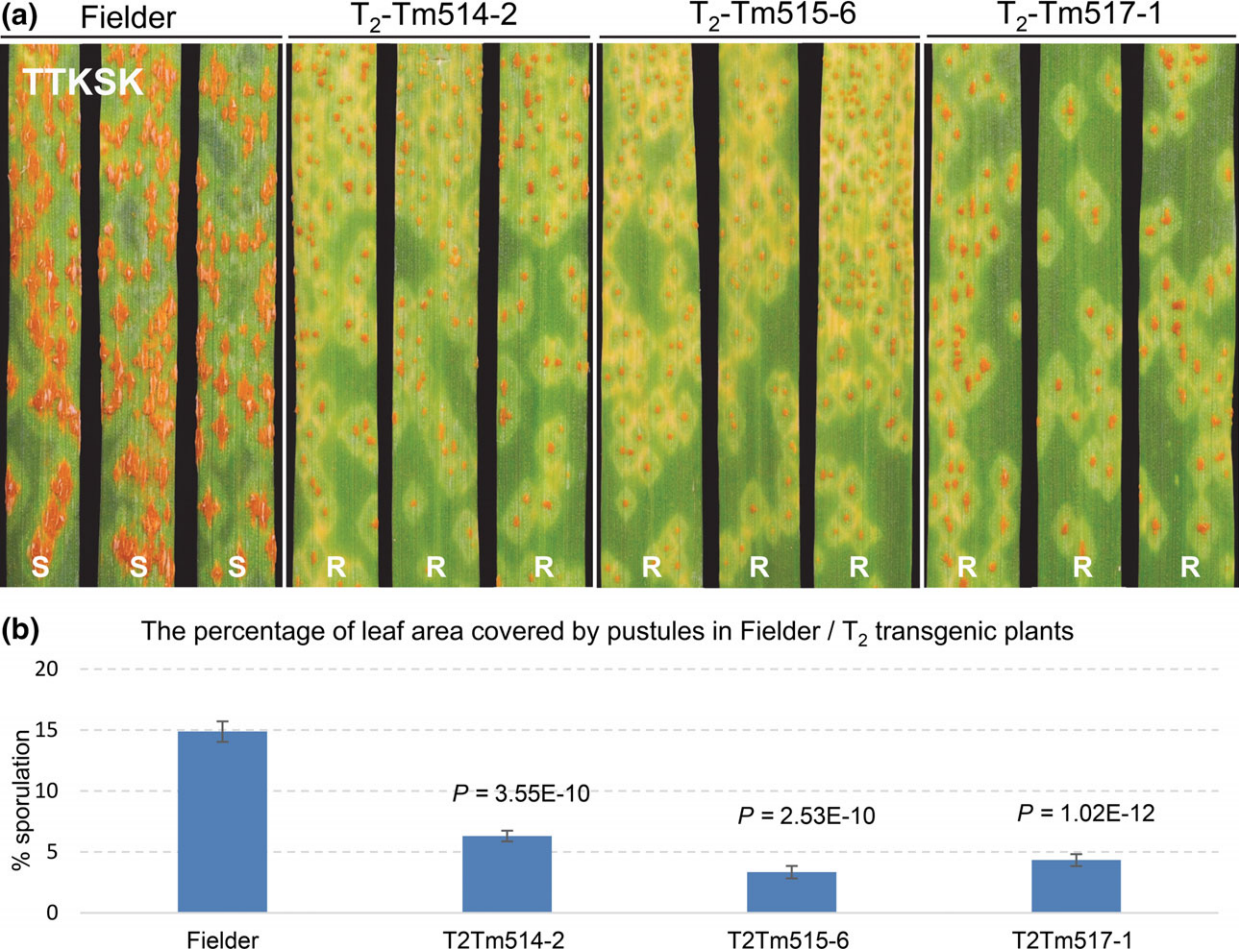
Gene *TmNLR1* confers resistance when transferred into the susceptible wheat variety Fielder. (a) Reactions to *Pgt* race TTKSK (isolate 04KEN156/04) in Fielder control and three transgenic families T_2_Tm514‐2, T_2_Tm515‐6 and T_2_Tm517‐1. S, susceptible; R, resistant. (b) The average percentage of the leaf area covered by *Pgt* pustules was measured using the software ASSESS v.2. More than 20 independent T_2_ plants were evaluated. Error bars are standard errors of the mean.

Roughly 25 T_2_ plants from each transgenic event and the untransformed control Fielder were challenged with *Pgt* race TTKSK (isolate 04KEN156/04). All plants from T_2_ transgenic families T_2_Tm514‐2 and T_2_Tm517‐1 fixed for the transgene showed high levels of resistance (Figure 2a), whereas resistance in Tm515‐6 T_2_ plants perfectly co‐segregated with the presence of the transgene (Figure S5). Measures of the percentage of leaf area covered by *Pgt* pustules was significantly lower (*P* < 0.0001) in the resistant transgenic plants of these three families (ranging from 1.3% to 9.2%) than in the non‐transgenic Fielder control (ranging from 10.3% to 24.6%) (Figure 2b). The progeny of the other five transgenic families displayed susceptible reactions similar to Fielder in all plants suggesting that the resistance gene was broken or damaged during the bombardment insertion. These transgenic families were discarded for further analysis.

To test if the transgenic plants had the same resistance profile as the natural *SrTm5* gene in monogenic line TmR54‐3, we inoculated transgenic family T_2_Tm514‐2 (homozygous for the transgene) with another two *Pgt* races RTJRM and 21C3CTTTM, which are virulent on *SrTm5* in *T*. *monococcum*. Plants from T_2_Tm514‐2 showed susceptible reactions similar to Fielder when challenged with *SrTm5*‐virulent races RTJRM and 21C3CTTTM (Figure S6) but were resistant when challenged with TTKSK (Figure 2), suggesting similar race specificity between the transgene and natural *SrTm5* in *T*. *monococcum*.

Taken together, the map‐based cloning and transgenic complementation results demonstrate that *SrTm5* is an allele of the cloned gene *Sr22*. Based on its different resistance profiles (Table S4), we designated the R1 (Schomburgk/PI 660256) and R4 (PI 190945) haplotypes as allele *Sr22a*, and *SrTm5* as allele *Sr22b*. This nomenclature has been approved by the Catalogue of Gene Symbols for wheat.

### Effect of *Pgt* inoculation on transcript levels of *Sr22b*

**Figure 3:**
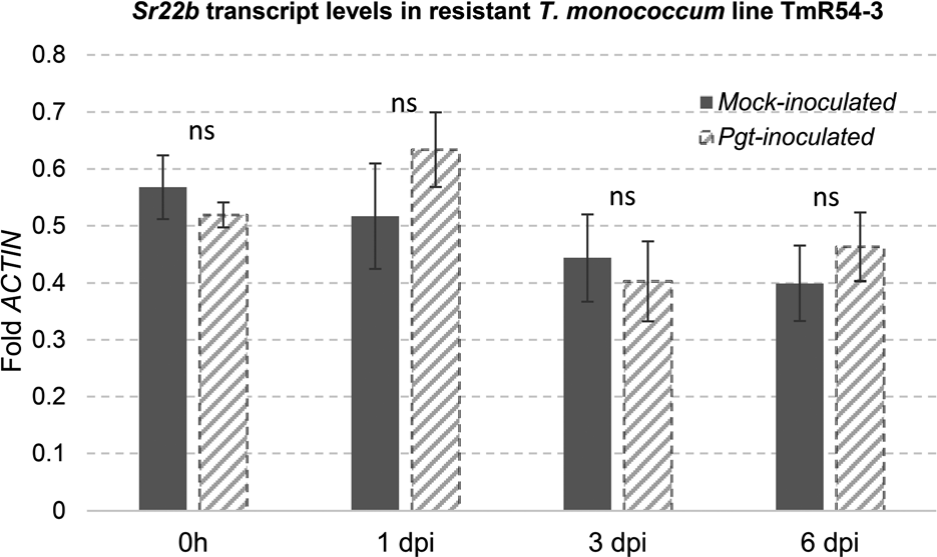
Transcript levels of *Sr22b* in mock‐inoculated and *Pgt*‐inoculated *T*. *monococcum* plants. Leaves were collected from *Sr22b* monogenic line TmR54‐3 at four time points: 0 h, 1 dpi, 3 dpi and 6 dpi. Plants were grown in growth chambers at 22 °C day/20 °C night with 16 h light/8 h dark. Transcript levels were expressed as fold‐*ACTIN* (*n* = 4). ns = not significant; Error bars are standard errors of the mean.

We analysed *Sr22b* transcript levels relative to *ACTIN* in the monogenic line TmR54‐3 by qRT‐PCR. We found no significant transcriptional differences between plants inoculated with *Sr22b*‐avirulent *Pgt* race 34PKUSC and mock inoculated with water at 1, 3 and 6 days post inoculation (dpi) (Figure 3), suggesting that *Sr22b* is not induced by the presence of the *Pgt* pathogen. We also compared the transcript levels of *Sr22a* in *T*. *monococcum* accession PI 190945 and *Sr22b* in *T*. *monococcum* line TmR54‐3 before inoculation and found no significant differences between them (Figure S7).

### *Sr22b* is present only in *T*. *monococcum*

The dominant marker *TM5TF2R2* was designed based on the special polymorphism (the insertion of repetitive sequence in the second intron) that differentiates *Sr22b* from the cloned *Sr22*‐resistant haplotypes and all susceptible alleles. The forward primer was designed in the second intron and the reverse primer in the inserted retrotransposon. Amplification with PCR marker *TM5TF2R2* at an annealing temperature of 60 °C generates an amplicon of 673 bp only when the gene *Sr22b* is present (Figure S8). Using this marker, we evaluated a collection of 165 wheat accessions, including 89 accessions of *T*. *monococcum*, 23 of *T. turgidum* and 53 of *T. aestivum*. PCR products were present only in 13 (14.6%) of the *Triticum monococcum* accessions but were absent in all tetraploid and hexaploid wheat lines tested in this study (Table S5). These observations were consistent with Sanger sequencing results using two pairs of primers *TM5AF6R8* and *TM5AF4R4* (Table 1), which were designed to amplify the LRR region of *Sr22*. The 13 *T*. *monococcum* accessions with the retrotransposon insertion, all carry the *Sr22b* haplotype in the LRR coding region, whereas all the other accessions have different haplotypes in the coding region and lack the retrotransposon insertion.

We then used the *TM5TF2R2* marker to explore the presence of *Sr22b* in *T*. *monococcum* accessions PI 355538, PI 362610 and PI 377668 from the Balkans (Table S6), which were previously postulated to carry an unknown *Pgt* resistance gene different from *Sr21* based on their different resistance reactions to races BCCBC and MCCFC (Chen *et al*., 2018b). We found that these three lines have *Sr22b*, which can explain their resistance to *Pgt* race MCCFC but susceptibility to BCCBC. This was confirmed by phenotyping 48 plants with race 34PKUSC in three F_2_ populations derived from crosses between PI 355538, PI 362610 and PI 377668 and the susceptible accession PI 272557. Genotyping with marker *TM5TF2R2* showed that all plants in which the 673‐bp fragment was amplified were resistant, whereas all plants without PCR products were susceptible. Moreover, we sequenced the coding regions of *Sr22* from PI 355538, PI 362610 and PI 377668, and found that they were all 100% identical to *Sr22b* in PI 306540. These results confirmed that the resistance to MCCFC and 34PKUSC in these accessions was conferred by *Sr22b*.

### Introgression of *Sr22b* into hexaploid wheat background

**Figure 4:**
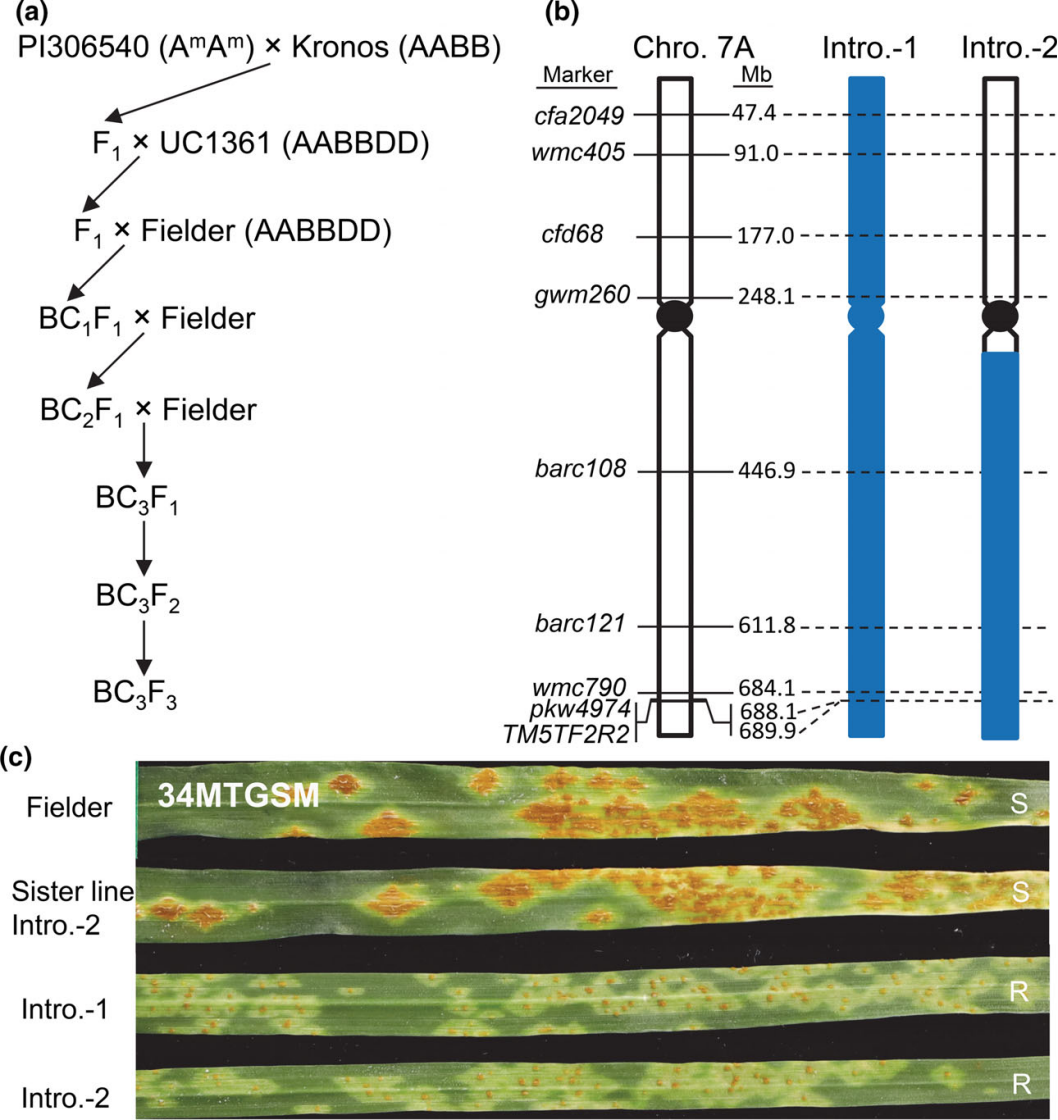
Introgression of *Sr22b* into common wheat background. (a) The procedure for the production of *Sr22b* introgression lines. Markers *TM5TF2R2* and *pkw4974* (digested with *Hae*III; Table 1) were used for confirming the presence of *T*. *monococcum* chromatin. (b) Markers on chromosome 7A were used to determinate the length of the introgression segments. The physical locations of polymorphic markers were based on the Chinese Spring reference genome Refseq v1.0. Blue rectangles indicate *T*. *monococcum* chromatin. (c) Infection types from Fielder control, introgression lines Intro.‐1 and Intro.‐2 and its sister line (named ‘Sister line Intro.‐2’) lacking *Sr22b*. BC_3_F_3_ plants were challenged with *Pgt* race 34MTGSM. S, susceptible; R, resistant.

Figure 4a describes the crosses involved in the generation of the *Sr22b* introgression into hexaploid wheat. The diagnostic marker *TM5TF2R2* and the closely linked CAPS marker *pkw4974* (Table 1) were used for monitoring the presence of *T*. *monococcum* chromatin during backcrosses and for the final selection of BC_3_F_2_ plants homozygous for *Sr22b*. We confirmed the absence of stem rust resistance genes *Sr13*, *Sr60*, *Sr21* and *SrTm4* from the parental lines using diagnostic or closely linked markers (Briggs *et al*., 2015; Chen *et al*., 2020; Chen *et al*., 2018b; Zhang *et al*., 2017).

To determine the size of the 7A^m^ chromosome region introgressed into hexaploid wheat, we first screened lines PI 306540, Kronos and Fielder for polymorphisms using 23 SSR markers distributed along chromosome 7A. We obtained seven polymorphic markers (Table 1) and determined their physical locations in the Chinese Spring reference genome (Refseq v1.0; Figure 4b). We genotyped 13 BC_3_F_1_ plants with markers *TM5TF2R2* and *pkw4974*, and detected five plants with the 7A^m^L introgression. BC_3_F_1_ plants 1, 3, 4 and 5 carried the 7A^m^L alleles for all the tested markers extending from 47.4 Mb to 689.9 Mb suggesting that they are disomic 7A^m^ (7A) substitution lines (Intro. 1 henceforth). The *T*. *monococcum* segment in plant number 2 extended from 446.9 Mb (*barc108*) to 689.9 Mb (*TM5TF2R2*), indicating a translocation of part of the long arm (referred hereafter as Intro.2, Figure S9). All these plants exhibited good levels of fertility when self‐pollinated.

Homozygous BC_3_F_3_ plants from these introgression lines challenged with Chinese *Pgt* race 34MTGSM showed good levels of resistance, whereas the recurrent parent Fielder and its sister line lacking *Sr22b* were completely susceptible (Figure 4c). Small amounts of BC_3_F_3_ seeds from the introgression lines are available by request from the senior authors. After the seed is increased, it will be deposited in the National Small Grain Collection in the United States and in the Chinese Crop Germplasm Resources Information System (CGRIS) in China.

## Discussion

In this study, we confirmed that *SrTm5* is a new allele of *Sr22*, officially designated as *Sr22b*. The stem rust resistance gene *Sr22* was previously identified to encode a coiled‐coil nucleotide‐binding leucine‐rich repeat protein, which confers broad‐spectrum resistance to commercially important *Pgt* races, including the Ug99 race group (Steuernagel *et al*., 2016). *Sr22b* and *Sr22a* both confer strong levels of resistance to *Pgt* races TTKSK (Ug99), TTKST, MCCFC, 34MTGSM and 34PKUSC, but differ in that *Sr22b* is susceptible to races BCCBC, 21C3CTTTM, RTJRM, QFCSC, TRTTF and TTTTF and *Sr22a* is not (Table S4). These results suggest that the *Sr22a* allele (R1 and R4 haplotypes) confers a broader resistance to tested *Pgt* races than *Sr22b* (Table S4). We currently do not know whether the other four *Sr22‐*resistant haplotypes (R2, R3, R5 and R6, Figure S3) have different resistance profiles because monogenic lines are not available for these haplotypes.

The different *Pgt* resistance profiles of *Sr22a* and *Sr22b* were associated with more than 30 polymorphisms, located mostly within the LRR region (Figure S3). The LRR domain of plant NLR genes is known to play a major role in pathogen recognition specificity, and diversifying selection drives higher levels of sequence variation (Dodds *et al*., 2006; Jiang *et al*., 2007; Krasileva and Dahlbeck, 2010). The different resistance profiles of *Sr22a* and *Sr22b* provide a useful tool to study the recognition mechanisms between Sr22 and the corresponding Avr proteins.

Insertions of large retrotransposons into functional genes is not a rare phenomenon in wheat, and can result in loss of function if inserted in the coding region. Insertions in introns may or may not have functional effects in the expression of the gene. For example, the gene *Zfp69* is disrupted by a inserted retrotransposon in its intron, which generates a truncated mRNA (Scherneck *et al*., 2009) and insertion of retrotransposons into the intron of Maize *waxy* gene caused alternative splicing (Varagona and Purugganan, 1992). Unlike these genes, the large retrotransposon insertion in the intron of *Sr22b* did not affect its expression levels or function (Figure S7). We used this distinctive retrotransposon insertion in *Sr22b* to develop a diagnostic marker for this allele.

The complete coding region, UTRs and the inserted retrotransposon of *Sr22b* were too large to clone into a binary vector for *Agrobacterium*‐mediated transformation, so we performed biolistic transformation using the circular BAC plasmid Tm84C1, which carries the 103.4‐kb genomic fragment of PI 306540 and the 8.1‐kb vector backbone sequence. Transformation with DNA fragments or circular plasmids larger than 100 kb has been previously reported in several plant species, such as tobacco (Wang *et al*., 2015), potato (Ercolano *et al*., 2004) and rice (Wang *et al*., 2015), but we are not aware of similar examples in wheat. Very large genes transformed by bombardment can be broken and disrupted (Liu *et al*., 2019; Makarevitch and Svitashev, 2003; Svitashev *et al*., 2002), which can explain the five confirmed transformation events that were susceptible to *Pgt*.

Fortunately, three independent events showed strong levels of resistance after infection with *Pgt* race TTKSK, indicating that the whole *Sr22b* gene was integrated into the plant genome in these three transgenic lines. We observed a significant segregation distortion against the transgene both in T_1_ and T_2_ families (Table S3), but the distortion was not that strong, and we were able to recover plants homozygous for the different transformation events that showed stable resistance to *Pgt*.

*Sr22b* was successfully introgressed into the common wheat variety Fielder, where it conferred good levels of resistance to *Pgt* (Figure 4). However, the sizes of the *T*. *monococcum* introgression are quite large, including the whole 7A^m^ chromosome or most of the long arm of chromosome 7A^m^ (Figure S9). More work will be needed to reduce the length of the introgressed *T*. *monococcum* chromosome segment to minimize potential linkage drag. Fortunately, recombination between the A and A^m^ chromosomes can be restored to normal levels through using the *ph1b* mutation (Dubcovsky *et al*., 1995). The diagnostic marker for *Sr22b* and the flanking SSR markers (Figure S9; Table 1) will be useful tools to develop shorter *T*. *monococcum* introgression lines carrying *Sr22b*.

*Sr22b* is only present in few cultivated *T*. *monococcum* accessions but absent in all tested polyploid wheats, indicating that it has the potential to improve Ug99 resistance in a wide range of modern wheat cultivars. However, since *Sr22b* is susceptible to several *Pgt* races, it would be necessary to combine with other resistance genes to provide a broader virulence spectrum. *Sr* genes that are susceptible to race TTKSK but effective to other *Pgt* races could be considered as candidates for combination with *Sr22b*. Examples of these complementary genes include *Sr60* (Chen *et al*., 2020), *Sr8155B1* (Nirmala *et al*., 2017), *Sr_TRTTF* (Hiebert *et al*., 2017) and *Sr9e* (Olivera *et al*., 2012).

The cloning of *SrTm5* demonstrated that it is a new allele of *Sr22* and brings close to completion the characterization of all previously mapped stem rust resistance genes in *T*. *monococcum* (*Sr21*, *Sr22*, *Sr35* and *Sr60*). The only mapped gene that has not been cloned yet is the recessive resistance gene *SrTm4* (Briggs *et al*., 2015). This information expands our understanding of the role of different stem rust resistance gene combinations in the adaptation of diploid wheat to this damaging rust pathogen and provides an entry point to understand the recognition specificity of different *Sr22* alleles to different *Pgt* races and effectors. From a practical point of view, the identification of *Sr22b*, its transfer to hexaploid wheat and the reliable diagnostic marker developed in this study provide a useful tool to diversify the *Sr* genes deployed in modern wheat breeding programmes.

## Methods

### *T*. *monococcum* materials and mapping populations

As a source of *SrTm5*, we used *T*. *monococcum* subsp. *monococcum* accession PI 306540, which was collected in Romania and that was previously shown to express the high levels of resistance to different *Pgt* races (Rouse and Jin, 2011a). PI 306540 was crossed with *T*. *monococcum* cultivated accession PI 272557, which does not carry any known *Sr* genes (Rouse and Jin, 2011b). Since PI 306540 carries multiple *Sr* genes, we selected F_5_ families segregating only for *SrTm5* from the cross PI 306540 × PI 272557 (Chen *et al*., 2018a). A total of 2264 segregating gametes were used to construct a high‐density genetic map of *SrTm5*. From this population, we selected the monogenic F_5_ line TmR54‐3 homozygous for *SrTm5* (without any of the other resistance genes) and the sister control line TmS57‐57 carrying no stem rust resistance gene.

A collection of 92 accessions of *T*. *monococcum*, 23 of *T. turgidum* and 53 of *T. aestivum* obtained from the US Department of Agriculture National Small Grains Collection (USDA‐NSGC, https://npgsweb.ars‐grin.gov/gringlobal/search) or the Chinese Crop Germplasm Resources Information System (CGRIS, http://www.cgris.net/cgris_english.html) were used to test the presence/absence of *SrTm5*.

### Stem rust evaluation

Previously, infection types of *SrTm5* to multiple *Pgt* races were reported, including TTKSK (isolate 04KEN156/04), TTKST (06KEN19v3), MCCFC (59KS19), QTHJC (75ND717C), QFCSC (06ND76C), SCCSC (09ID73‐2), TTTTF (01MN84A‐1‐2), TRTTF (06YEM34‐1) and TKTTF (13ETH18‐1 and 13GER15‐1) (Chen *et al*., 2018a). In this study, stem rust seedling tests were carried out in three institutions: Peking University Institute of Advanced Agricultural Sciences, Weifang, China; USDA‐ARS Cereal Disease Laboratory, Minnesota, USA; and University of California, Davis, USA. Selected sister lines TmR54‐3 and TmS57‐57 were re‐evaluated with race TTKSK (04KEN156/04). To expand the resistance profile of *SrTm5*, we also evaluated these lines with North American race BCCBC (09CA115‐2) and Chinese races 34MTGSM (20GSA1), 21C3CTTTM (20GH13), RTJRM (mutant strain, 20IAS11) and 34PKUSC (19IAS08) (Li *et al*., 2016, 2018; Zhao *et al*., 2015). The origin and virulence/avirulence profiles of these *Pgt* races are presented in supplemental Table S1. Procedures for inoculation and scoring infection types (ITs) were as previously reported (Rouse *et al*., 2011; Stakman and Stewart, 1962).

For plants carrying critical recombination events in the high‐density map, we preformed progeny tests including at least 25 progenies. These plants were inoculated with Chinese *Pgt* race 34PKUSC, and the percentage of the leaf area covered with pustules was estimated using the software ASSESS version 2.0 (American Phytopathological Society, St Paul, MN, USA) as reported previously (Lamari, 2008).

### BAC library screening and sequencing

A non‐gridded BAC library from PI 306540 with roughly 5× genome equivalents was available at the Wheat Molecular Genetics Laboratory, University of California, Davis (Chen *et al*., 2020). A PCR screening was performed using increasingly diluted library samples following the manufacturer’s instruction (Amplicon Express Inc., Pullman, WA). Screening of the BAC library with PCR markers *pkw4995*, *Tm5F3R4*, *pkw4997* and *pkw4999* yielded two positive BAC clones Tm84C1 and Tm2677. High quality BAC DNAs were extracted using Qiagen Large‐Construct Kits (Qiagen, Hilden, Germany) and sequenced with Wideseq at Purdue University (https://purdue.ilabsolutions.com/landing/808). Repetitive elements were identified and annotated using the Cereal Repeat Sequences Database (https://wheat.pw.usda.gov/ITMI/Repeats/blastrepeats3.html). Candidate genes were annotated using the published reference genomes (The International Wheat Genome Sequencing Consortium, 2018; Walkowiak *et al*., 2020), and confirmed using the BLASTN/BLASTX searches available at National Center for Biotechnology Information (NCBI, https://www.ncbi.nlm.nih.gov/). Expression profiles were determined with the Wheat Expression Browser (expVIP, http://www.wheat‐expression.com/).

### Wheat transformation

Bacterial artificial chromosome clone Tm84C1 containing 103 429 bp of *T*. *monococcum* PI 3065040 genomic sequence (GenBank accession MZ327628) was cloned into vector pCC1BAC (8128 bp). The cloned *T*. *monococcum* region carries complete genes *TmB3* and *TmNLR1* and a partial sequence of gene *TmPPR1* (missing 30% of the distal coding region). Biolistic transformation was performed using a PDS1000/He particle bombardment system (Bio‐Rad, Hercules, CA, USA). The cloned BAC Tm84C1 was co‐transformed with plasmid pAHC20, which carries *bialaphos* (*BAR*) selectable marker gene. BAC DNAs were mixed in a 1 : 1 (1 : 1 for BAC DNA and pAHC20) molar ratio prior to bombardment. Transformation was performed using the Ug99‐susceptible spring wheat variety Fielder by biolistic bombardment as described previously (Zhang *et al*., 2015).

Positive transgenic plants were identified using dominant or codominant PCR markers *Tm5F3R4*, *TM5TF2R2* and *TM5TF3R3* (Table 1). Expression levels of *TmNLR1* in transgenic plants were assessed by quantitative real‐time PCR (qRT‐PCR) with primer pairs *HL‐F61R60*. About 25 T_2_ transgenic seeds from each transgenic event were germinated and tested for their responses to *Pgt* race TTKSK (Ug99).

### qRT‐PCR analysis

Plants from *SrTm5* monogenic line TmR54‐3 were mock inoculated or *Pgt* inoculated in two independent chambers under the same environmental condition: 22 °C day/20 °C night and 16 h light/8 h dark. Total RNAs were extracted from leaves of different plants collected immediately after inoculation (0 h) and 1, 3 and 6 days post inoculation (dpi) using Spectrum Plant Total RNA Kit (Sigma‐Aldrich, Saint Louis, MO, USA). First‐strand cDNA was synthesized using the Applied Biosystems™ High‐Capacity cDNA Reverse Transcription Kits. qRT‐PCR reactions were performed on a QuantStudio™ 5 real‐time PCR system (Thermo Fisher Scientific, Inc., Waltham, MA, USA) using Fast SYBR GREEN reagents. PCR primers *A120F6R6* (Table 1, 97% efficiency) were used to evaluate the effect of *Pgt* inoculation on *SrTm5*. Transcript levels were determined in four biological replicates and expressed as fold‐*ACTIN* levels as described previously (Pearce and Vanzetti, 2013).

### Introgression of *T*. *monococcum* segments carrying *SrTm5* into hexaploid wheat

The diploid wheat accession PI 306540 (A^m^A^m^) was used for transferring *T*. *monococcum* gene *SrTm5* to hexaploid wheat variety Fielder using *T. durum* wheat variety Kronos (AABB) as bridging species (The, 1973). The F_1_ triploid plants from the cross of PI 306540 × Kronos were crossed with hexaploid wheat variety Clear White (UC1361), and the resulting F_1_ plants were backcrossed three times to the recurrent spring common wheat line Fielder. PCR markers *TM5TF2R2* and *pkw4974* (Table 1) were used to validate the presence of the introgressed *T*. *monococcum* segments during backcrossing. Five BC_3_F_1_ plants carrying alien chromosome segments were self‐pollinated and characterized with 23 simple sequence repeat (SSR) markers across chromosome 7A to analyse the length of introgressed *T*. *monococcum* segments. Subsequently, we selected BC_3_F_2_ plants homozygous for the introgressed *T*. *monococcum* segment to generate seeds. The resulting progeny were inoculated with *Pgt* race 34MTGSM.

## Conflicts of interest

The authors declare that they have no conflict of interests.

## Author contributions

JL and MNR performed most of the experimental work; YW and CG designed the transgenic experiments. BL performed the biolistic transformation and obtained T_1_ seeds. LeiH contributed qRT‐PCR and filled the gaps of BAC sequence; HnaL contributed primers development; TL performed part of the phenotyping experiments; WZ created the mapping population and contributed sequence analyses. SC analysed the data and wrote the first version of the manuscript. YW, SC and JD proposed and supervised the project, obtained the funding and generated the final version of the paper. All authors revised the manuscript and provided suggestions.

## Supporting information


**Figure** **S1** Reactions to six *Pgt* races 34PKUSC, 34MTGSM, TTKSK, BCCBC, 21C3CTTTM and RTJRM.
**Figure** **S2** Semi‐quantitative PCR products from markers *4997QF2R2* (260 bp, *TraesCS7A02G499700*), *4998QF5R5* (272 bp, *TraesCS7A02G499800*) and *ACTINF1R1* (*ACTIN*).
**Figure** **S3** SrTm5 protein sequence analysis. Multiple sequence alignment between SrTm5 and reported Sr22‐resistant and susceptible protein sequences (Steuernagel *et al*. 2016).
**Figure S4** Transcript levels of *TmNLR1* in transgenic T_1_ families (three positive plants per event, *n* = 3).
**Figure** **S5** Reactions to *Pgt* race TTKSK (Ug99) in transgenic family T_2_Tm515‐6.
**Figure** **S6** Transgenic family T_2_Tm514‐2 homozygous for the transgene were inoculated with two *SrTm5‐*virulent *Pgt* races RTJRM and 21C3CTTTM.
**Figure S7** Transcript levels and infection types of *Sr22a* and *Sr22b* in *T. monococcum* background.
**Figure S8** PCR products from the *Sr22b* diagnostic marker *TM5TF2R2*.
**Figure** **S9** Markers across chromosome 7A were used to analyse the length of introgressed *T. monococcum* segments.
**Table S1** Avirulence/virulence formulae of *Pgt* races, and their responses to *SrTm5*.
**Table** **S2** Comparison of SrTm5 protein with polymorphisms that discriminate perfectly between Sr22‐susceptible and ‐resistant haplotypes from Steuernagel *et al*. (2016).
**Table S3** Segregation ratios in T_1_ and T_2_ transgenic families detected using PCR markers *Tm5F3R4*, *TM5TF2R2* and *TM5TF3R3* (Table 1).
**Table** **S4** Resistance profiles of *Sr22b* (=*SrTm5*) and *Sr22a* (haplotypes R1 and R4) to multiple *Pgt* races.
**Table** **S5** A collection of 92 accessions of *T. monococcum*, 23 of *T. turgidum* and 53 of *T. aestivum* was used to test the presence of *Sr22b*.
**Table** **S6** Geographic distribution of *T. monococcum* accessions, and their reactions against *Pgt* races TTKSK, MCCFC and 34PKUSC.
