# Supplementary material for: Identification and characterization of Sr22b, a new allele of the wheat stem rust resistance gene Sr22 effective against the Ug99 race group
Source: Plant Biotechnol J. 2021 Nov 6;20(3):554–63. doi: 10.1111/pbi.13737 (PMC8882774; doi:10.1111/pbi.13737)
Supplement: Supplementary file 1 — Figure S1 Reactions to six Pgt races 34PKUSC, 34MTGSM, TTKSK, BCCBC, 21C3CTTTM and RTJRM. Figure S2 Semi‐quantitative PCR products from markers 4997QF2R2 (260 bp, TraesCS7A02G499700), 4998QF5R5 (272 bp, TraesCS7A02G499800) and ACTINF1R1 (ACTIN). Figure S3 SrTm5 protein sequence analysis. Multiple sequence alignment between SrTm5 and reported Sr22‐resistant and susceptible protein sequences (Steuernagel et al. 2016). Figure S4 Transcript levels of TmNLR1 in transgenic T1 families (three positive plants per event, n = 3). Figure S5 Reactions to Pgt race TTKSK (Ug99) in transgenic family T2Tm515‐6. Figure S6 Transgenic family T2Tm514‐2 homozygous for the transgene were inoculated with two SrTm5‐virulent Pgt races RTJRM and 21C3CTTTM. Figure S7 Transcript levels and infection types of Sr22a and Sr22b in T. monococcum background. Figure S8 PCR products from the Sr22b diagnostic marker TM5TF2R2. Figure S9 Markers across chromosome 7A were used to analyse the length of introgressed T. monococcum segments. Table S1 Avirulence/virulence formulae of Pgt races, and their responses to SrTm5. Table S2 Comparison of SrTm5 protein with polymorphisms that discriminate perfectly between Sr22‐susceptible and ‐resistant haplotypes from Steuernagel et al. (2016). Table S3 Segregation ratios in T1 and T2 transgenic families detected using PCR markers Tm5F3R4, TM5TF2R2 and TM5TF3R3 (Table 1). Table S4 Resistance profiles of Sr22b (=SrTm5) and Sr22a (haplotypes R1 and R4) to multiple Pgt races. Table S5 A collection of 92 accessions of T. monococcum, 23 of T. turgidum and 53 of T. aestivum was used to test the presence of Sr22b. Table S6 Geographic distribution of T. monococcum accessions, and their reactions against Pgt races TTKSK, MCCFC and 34PKUSC. [file PBI-20-554-s001.pdf]

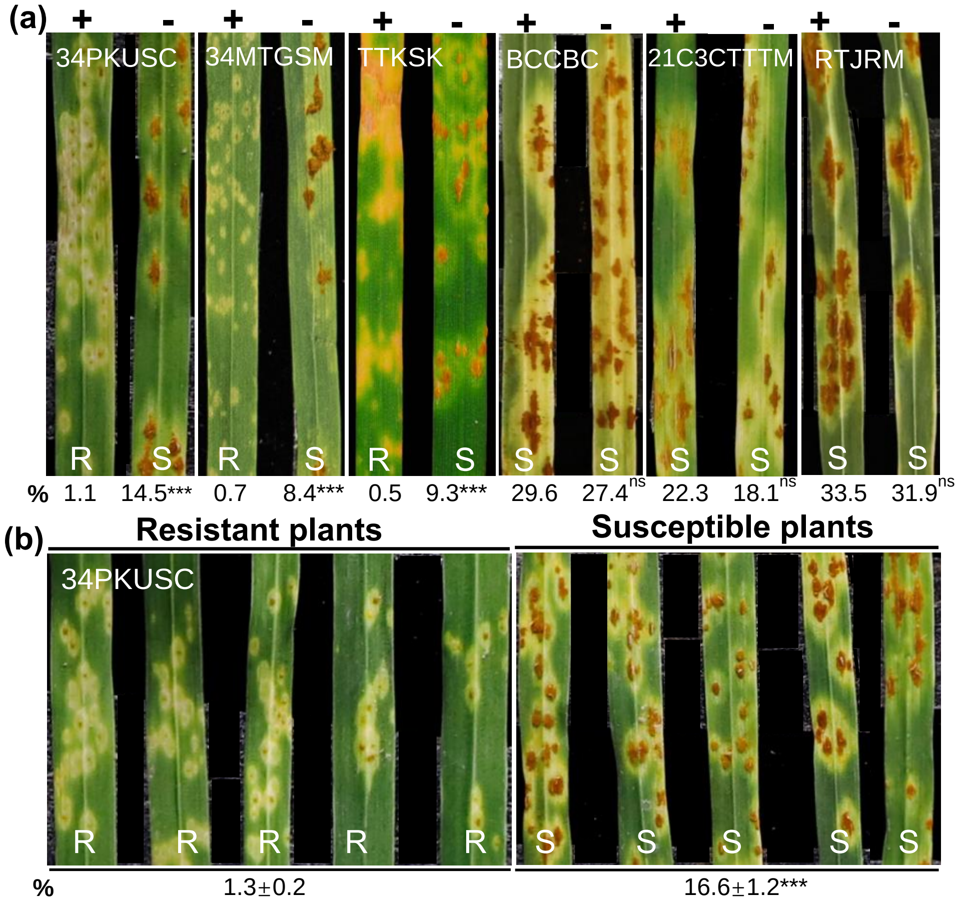


**Figure S1** Reactions to six *Pgt* races 34PKUSC, 34MTGSM, TTKSK, BCCBC, 21C3CTTTM and RTJRM. (a) Infection types in *Triticum monococcum* $F_5$ lines TmR54-3 homozygous for *SrTm5* and its sister line TmS57-57 carrying no stem rust resistance gene. (b) Infection types on segregating resistant and susceptible plants when inoculated with race 34PKUSC. Numbers listed below leaves are average percentage of leaf area covered by *Pgt* pustules (n = 6). +, TmR54-3 (with *SrTm5*); -, TmS57-57 (without *SrTm5*); R, resistant; S, susceptible; ns= not significant ($P$ >0.05), ***, $P$ < 0.001.

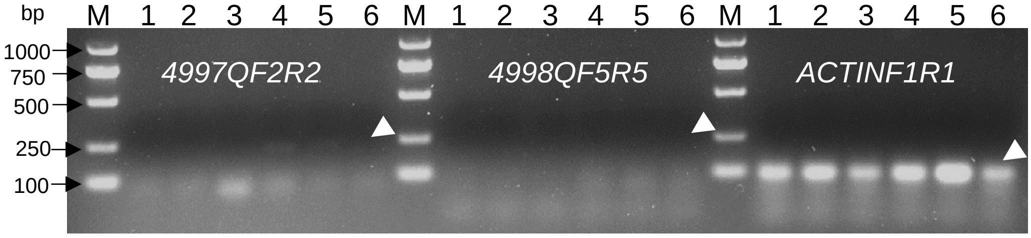


**Figure S2** Semi-quantitative PCR products from markers *4997QF2R2* (260 bp, *TraesCS7A02G499700*), *4998QF5R5* (272 bp, *TraesCS7A02G499800*) and *ACTINF1R1* (*ACTIN*). RNAs were extracted from *Pgt*-inoculated leaves from *SrTm5* monogenic line TmR54-3. cDNAs were synthesized using the Applied Biosystems™ High-Capacity cDNA Reverse Transcription Kits. *ACTIN* was used as endogenous control. Six independent plants were evaluated. 1-3, samples collected at 3 dpi; 4-6, samples collected at 6 dpi; M, markers; Arrowheads indicate the expected band sizes.

| | | | |
|---|---|---|---|
| SCHOMBURGK_R1 | 1 | MAEVLVSASTGAMGSLLRKLGAMLTDEYKLLKNVRGDIKFLKDELEVMCAFLLKMSDVEEPDEPTKLRVTAVREMSYKIEDNIDKFMVLVEQEHGSSCSEAAHGVAKLMDKCKNLLPDIKARRRIAKEVKDIKKEIKDVSDRFSRYKIDDSSSSMP-AKD | 160 |
| IG44855_R2 | 1 | MAEVLVSASTGAMGSLLRKLGAMLTDEYKLLKNVRGDIKFLKDELEVMCAFLLKMSDVEEPDEPTKLRVTAVREMSYKIEDNIDKFMVLV--EHGSSCSEAAHGVAKLMDKCKNLLPDIKARRRIAKEVKDIKKEIKDVSDRFSRYKIDDSSSSMP-AKD | 160 |
| IG44921_R3 | 1 | MAEVLVSASTGAMGSLLRKLGAMLTDEYKLLKNVRGDIKFLKDELEVMCAFLLKMSDVEEPDEPTKLRVTAVREMSYKIEDNIDKFMVLV--EHESSCSEAAHGVAKLMDKCKNLLPDIKTRRRIAKEVKDIKKEIKDVSDRFSRYKIDESSSSMP-AKG | 160 |
| PI190945_R4 | 1 | MAEVLVSASTGAMGSLLRKLGAMLTDEYKLLKNVRGDIKFLKDELEVMCAFLLKMSDVEEPDEPTKLRVTAVREMSYKIEDNIDKFMVLV--EHESSCSEAAHGVAKLMDKCKNLLPDIKTRRRIAKEVKDIKKEIKDVSDRFSRYKIDESSSSMP-AKG | 160 |
| PI289605_R5 | 1 | MAEVLVSASTGAMGSLLRKLGAMLTDEYKLLKNVRGDIKFLKDELEVMCAFLLKMSDVEEPDEPTKLRVTAVREMSYKIEDNIDKFMVLVEQEHGSSCSEAAHGVAKLMDKCKNLLPDIKARRRIAKEVKDIKKEIKDVSDRFSRYKIDDSSSSMP-AKD | 160 |
| PI330550_R6 | 1 | MAEVLVSASTGAMGSLLRKLGAMLTDEYKLLKNVRGDIKFLKDELEVMCAFLLKMSDVEEPDEPTKLRVTAVREMSYKIEDNIDKFMVLVEQEHGSSCSEAAHGVAKLMDKCKNLLPDIKARRRIAKEVKDIKKEIKDVSDRFSRYKIDDSSSSMP-AKD | 160 |
| **PI306540_SrTm5** | 1 | MAEVLVSASTGAMGSLLRKLGAMLTDEYKLLKNVRGDIKFLKDELEVMCAFLLKMSDVEEPDEPTKLRVTAVREMSYKIEDNIDKFMVLV--EHESSCSEAAHGVAKLMDKCKNLLPDIKTRRRIAKEVKDIKKEIKDVSDRFSRYKIDESSSSMP-AKG | 160 |
| DV92_S1 | 1 | MAEVLVSASTGAMGSLLRKLGAMLTDEYKLLKNVRGDIKFLKDELEVMCAFLLKMSDVEEPDEPTKLRVTAVREMSYKIEDNIDKFMVLV--EHESSSSE-AHGVTKLMDKCKNLLPDIKTRRRIAKEVKDIKKEIKDVSDRFLRYKIDDSSSSMPAAKD | 160 |
| IG44878_S2 | 1 | MAEVLVSASTGAMGSLLRKLGAMLTDEYKLLKNVRGDIKFLKDELEVMCAFLLKMSDVEEPDEPTKLRVTAVREMSYKIEDNIDKFMVLV--EHESSFSEAAHGVAKLMDKCKNLLPDIKTRRRIAKEVKDIKKEIKDVSDRFSRYKIDESSSSMP-AKG | 160 |
| PI355523_S3 | 1 | MAEVLVSASTGAMGSLLRKLGAMLTDEYKLLKNVRGDIKFLKDELEVMCAFLLKMSDVEEPDEPTKLRVTAVREMSYKIEDNIDKFMVLVEQEHGSSCSEAAHGVAKLMDKCKNLLPDIKARRRIAKEVKDIKKEIKDVSDRFSRYKIDDSSSSMP-AKD | 160 |
| PI573523_S4 | 1 | MAEVLVSASTGAMGSLLRKLGAMLTDEYKLLKNVRGDIKFLKDELEVMCAFLLKMSDVEEPDEPTKLRVTAVREMSYKIEDNIDKFMVLV--EHESSCSEAAHGVAKLMDKCKNLLPDIKTRRRIAKEVKDIKKEIKDVSDRFSRYKIDESSSSMP-AKG | 160 |
| WESTONIA_S5 | 1 | MAEVLVSASTGAMGSLLRKLGAMLTDEYKLLKNVRGDIKFLKDELEVMCAFLLKMSDVEEPDEPTKLRVTAVREMSYKIEDNIDKFMVLV--EHESSCSEAAHGVAKLMDKCKNLLPDIKTRRRIAKEVKDIKKQIKDVSDRFSRYKIDESSSSMP-AKE | 160 |
| PI272557_S6 | 1 | MAEVLVSASTGAMGSLLRKLGAMLTDEYKLLKNVRGDIKFLKDELEVMCAFLLKMSDVEEPDEPTKLRVTAVREMSYKIEDNIDKFMVLVEHE--SSCSEAAHGVAKLMDKCKNLLPDIKTRRRIAKEVKDIKKEIKDVSDRFSRYKIDESSSSMP-AKG | 160 |
| | | | |
| SCHOMBURGK_R1 | 161 | KVDPRLRAVYKDAAELVGIDGPKDELVKWLNEKEGQSLKSVSIVGYGGLGKTTLANQIRVNLGATFDCGAFVSISRKPDMKAILRSILSQITKKDDACSRLDDIQLIIDKIREFLQDTRYFIIIDDIWELGTWETLKCAFVKNTLGSRIIITTRIVDVAK | 320 |
| IG44855_R2 | 161 | KVDPRLRAVYKDAAELVGIDGPKDELVKWLNEKEGQSLKSVSIVGYGGLGKTTLANQIRVNLGATFDCGAFVSISRKPDMKAILRSILSQITKKDDACSRLDDIQLIIDKIREFLQDTRYFIIIDDIWELGTWETLKCAFVKNTLGSRIIITTRIVDVAK | 320 |
| IG44921_R3 | 161 | KVDPRLRAVYKDAAELVGIDGPKDELVKWLNEKEGQSLKSVSIVGYGGLGKTTLANQIRVNLGATFDCGAFVSISRKPDMKAILRSILSQITKKDEACSRLDDIQLIIDKIREFLQDTRYFIIIDDIWELGTWETLKCAFVKNTLGSRIIITTRIVDVAK | 320 |
| PI190945_R4 | 161 | KVDPRLRAVYKDAAELVGIDGPKDELVKWLNEKEGQSLKSVSIVGYGGLGKTTLANQIRVNLGATFDCGAFVSISRKPDMKAILRSILSQITKKDEACSRLDDIQLIIDKIREFLQDTRYFIIIDDIWELGTWETLKCAFVKNTLGSRIIITTRIVDVAK | 320 |
| PI289605_R5 | 161 | KVDPRLRAVYKDAAELVGIDGPKDELVKWLNEKEGQSLKSVSIVGYGGLGKTTLANQIRVNLGATFDCGAFVSISRKPDMKAILRSILSQITKKDDACSRLDDIQLIIDKIREFLQDTRYFIIIDDIWELGTWETLKCAFVKNTLGSRIIITTRIVDVAK | 320 |
| PI330550_R6 | 161 | KVDPRLRAVYKDAAELVGIDGPKDELVKWLNEKEGQSLKSVSIVGYGGLGKTTLANQIRVNLGATFDCGAFVSISRKPDMKAILRSILSQITKKDDACSRLDDIQLIIDKIREFLQDTRYFIIIDDIWELGTWETLKCAFVKNTLGSRIIITTRIVDVAK | 320 |
| **PI306540_SrTm5** | 161 | KVDPRLRAVYKDAAELVGIDGPKDELVKWLNEKEGQSLKSVSIVGYGGLGKTTLANQIRVNLGATFDCGAFVSISRKPDMKAILRSILSQITKKDEACSRLDDIQLIIDKIREFLQDTRYFIIIDDIWELGTWETLKCAFVKNTLGSRIIITTRIVDVAK | 320 |
| DV92_S1 | 161 | KVDPRLRAVYKDAAELVGIDGPKDELVKWLNEKEGQSLKSVSIVGYGGLGKTTLANQIRVNLGATFDCGAFVSISRKPDMKAILRSILSQITKKDEACPRLDDIQLIIDKIREFLQDTRYFIIIDDIWELGTWETLKCAFVKNTLGSRIIITTRIVDVAK | 320 |
| IG44878_S2 | 161 | KVDPRLRAVYKDAAELVGIDGPKDELVKWLNEKEGQSLKSVSIVGYGGLGKTTLANQIRVNLGATFDCGAFVSISRKPDMKAILRSILSQITKKDDACSRLDDIQLIIDKIREFLQDTRYFIIIDDIWELGTWETLKCAFVKNTLGSRIIITTRIVDVAK | 320 |
| PI355523_S3 | 161 | KVDPRLRAVYKDAAELVGIDGPKDELVKWLNEKEGQSLKSVSIVGYGGLGKTTLANQIRVNLGATFDCGAFVSISRKPDMKAILRSILSQITKKDDACSRLDDIQLIIDKIREFLQDTRYFIIIDDIWELGTWETLKCAFVKNTLGSRIIITTRIVDVAK | 320 |
| PI573523_S4 | 161 | KVDPRLRAVYKDAAELVGIDGPKDELVKWLNEKEGQSLKSVSIVGYGGLGKTTLANQIRVNLGATFDCGAFVSISRKPDMKAILRSILSQITKKDDACSRLDDIQLIIDKIREFLQDTRYFIIIDDIWELGTWETLKCAFVKNTLGSRIIITTRIVDVAK | 320 |
| WESTONIA_S5 | 161 | KVDPRLRAVYKDVTELVGIDGPKDELVKWLNEKEGQSLKSVSIVGYGGLGKTTLANQIRVNLRASFDCGAFVSISRKPDMKAILRSILSQITKKDDAYSRLDDIQLIIDKIREFLQDTRYFIIIDDIWELGTWETLKCAFVKNTLGSRIIITTRIVDVAK | 320 |
| PI272557_S6 | 161 | KVDPRLRAVYKDAAELVGIDGPKDELVKWLNEKEGQSLKSVSIVGYGGLGKTTLANQIRVNLGATFDCGAFVSISRKPDMKAILRSILSQITKKDDACSRLDDIQLIIDKIREFLQDTRYFIIIDDIWELGTWETVKCAFVKNTLGSRIIITTRIVDVAK | 320 |
| | | | |
| SCHOMBURGK_R1 | 321 | SCSPSSEDLVYEMKPLSEADSKKLFFKRIFGCEESCPDSLKEAANDILKKCRGLPLAINAISSVLVTTRETKEEWDRVRHSIRSSKVKSDIIETMNYILSLSYFDLPHHLRSCLLYLALFPEDQLIGRKRLVRRWISEGFIHGESGQDLMELGEEYFHQL | 480 |
| IG44855_R2 | 321 | SCSPSSEDLVYEMKPLSEADSKKLFFKRIFGCEESCPDSLKEAANDILKKCRGLPLAINAISSVLVTTRETKEEWDRVRHSIRSSKVKSDIIETMNYILSLSYFDLPHHLRSCLLYLALFPEDQLIGRKRLVRRWISEGFIHGESGQDLMELGEEYFHQL | 480 |
| IG44921_R3 | 321 | SCSPSSEDLVYEMKPLSEADSKKLFFKRIFGCEESCPDSLKEAANDILKKCRGLPLAINAISSVLVTTRETKEEWDRVRHSIRSSKVKSDIIETMNYILSLSYFDLPHHLRSCLLYLALFPEDQLIGRKRLVRRWISEGFIHGESGQDLMELGEEYFHQL | 480 |
| PI190945_R4 | 321 | SCSPSSEDLVYEMKPLSEADSKKLFFKRIFGCEESCPDSLKEAANDILKKCRGLPLAINAISSVLVTTRETKEEWDRVRHSIRSSKVKSDIIETMNYILSLSYFDLPHHLRSCLLYLALFPEDQLIGRKRLVRRWISEGFIHGESGQDLMELGEEYFHQL | 480 |
| PI289605_R5 | 321 | SCSPSSEDLVYEMKPLSEADSKKLFFKRIFGCEESCPDSLKEAANDILKKCRGLPLAINAISSVLVTTRETKEEWDRVRHSIRSSKVKSDIIETMNYILSLSYFDLPHHLRSCLLYLALFPEDQLIGRKRLVRRWISEGFIHGESGQDLMELGEEYFHQL | 480 |
| PI330550_R6 | 321 | SCSPSSEDLVYEMKPLSEADSKKLFFKRIFGCEESCPDSLKEAANDILKKCRGLPLAINAISSVLVTTRETKEEWDRVRHSIRSSKVKSDIIETMNYILSLSYFDLPHHLRSCLLYLALFPEDQLIGRKRLVRRWISEGFIHGESGQDLMELGEEYFHQL | 480 |
| **PI306540_SrTm5** | 321 | SCSPSSEDLVYEMKPLSEADSKKLFFKRIFGCEESCPDSLKEAANDILKKCRGLPLAINAISSVLVTTRETKEEWDRVRHSIRSSKVKSDIIETMNYILSLSYFDLPHHLRSCLLYLALFPEDQLIGRKRLVRRWISEGFIHGESGQDLMELGEEYFHQL | 480 |
| DV92_S1 | 321 | SCSPSSEDLVYEMKPLSEADSKKLFFKRIFGCEESCPDSLKEAANDILKKCRGLPLAINAISSLLATTRETKEEWDRVRHSIRSSKVKSDIIETMNYILSLSYFDLPHHLRSCLLYLALFPEDQLIERKRLVRRWISEGFIHGESGQALMELGEEYFHQL | 480 |
| IG44878_S2 | 321 | SCSPSSEDLVYEMKPLSEADSKKLFFKRIFGCEESCPDSLKEAANDILKKCRGLPLAINAISSLLVTTRETKEEWDRVRHSIRSSKVKSDIIETMNYILSLSYFDLPHHLRSCLLYLALFPEDQLIGRKRLVRRWISEGFIHGESGQDLMELGEEYFHQL | 480 |
| PI355523_S3 | 321 | SCSPSSEDLVYEMKPLSEADSKKLFFKRIFGCEESCPDSLKEAANDILKKCRGLPLAINAISSLLVTTRETKEEWDRVRHSIRSSKVKSDIIETMNYILSLSYFDLPHHLRSCLLYLALFPEDQLIGRKRLVRRWISEGFIHGESGQDLMELGEEYFHQL | 480 |
| PI573523_S4 | 321 | SCSPSSEDLVYEMKPLSEADSKKLFFKRIFGCEESCPDSLKEAANDILKKCRGLPLAINAISSLLVTTRETKEEWDRVRHSIRSSKVKSDIIETMNYILSLSYFDLPHHLRSCLLYLALFPEDQLIGRKRLVRRWISEGFIHGESGQDLMELGEEYFHQL | 480 |
| WESTONIA_S5 | 321 | SCSPSSEDLVYEMKPLSEADSKKLFFKRIFGCEESCPDSLKEAANDILKKCRGLPLAINAISSLLATTRETKEEWDRVRHSIHSSKVKSDIIETMNYILSLSYFDLPHHLRSCLLYLALFPEDQLIGRKRLVRRWISEGFIHGESGQDLMELGEEYFHQL | 480 |
| PI272557_S6 | 321 | SCSPSSEDLVYEMKPLSEADSKKLFFKRIFGCEESCPDSLKEAANDILKKCRGLPLAINAISSLLVTTRETKEEWDRVRHSIRSSKVKSDIIETMNYILSLSYFDLPHHLRSCLLYLALFPEDQLIGRKRLVRRWISEGFIHGESGQDLMELGEEYFHQL | 480 |

```
SCHOMBURGK_R1   481  VNRSLIQPGNIGYDGKAMYCRVHDTILDFLIDKSSEENMCTVLKKQCKPNGIVRRLSLMGNEDEEIVEQLDLSHARSITAFGDIKLLPSLGRSKCLRVLDLQDCDQLENHHIKDIERLYQLRYLDISSTGITELPRQIGELLYLETLV--AY-GLRELPE   640
IG44855_R2      481  VNRSLIQPGNIGYDGKAMYCRVHDTILDFLIDKSSEENMCTVLKKQCKPNGIVRRLSLMGNEDEEIVEQLDLSHARSITAFGDIKLLPSLGRSKCLRVLDLQDCDQLENHHIKDIERLYQLRYLDISSTGITELPRQIGELLYLETLV--AY-GLRELPE   640
IG44921_R3      481  VNRSLIQPGNIGYDGKAMYCRVHDTILDFLIDKSSEENMCTVLKKQCKPNGIVRRLSLMGNEDEEIVEQLDLSHARSITAFGDIKLLPSLGRSKCLRVLDLQDCDQLENHHIKDIERLYQLRYLDISSTGITELPRQIGELLYLETLV--AY-GLRELPE   640
PI190945_R4     481  VNRSLIQPGNIGYDGKAMYCRVHDTILDFLIDKSSEENMCTVLKKQCKPNGIVRRLSLMGNEDEEIVEQLDLSHARSITAFGDIKLLPSLGRSKCLRVLDLQDCDQLENHHIKDIERLYQLRYLDISSTGITELPRQIGELLYLETLV--AY-GLRELPE   640
PI289605_R5     481  VNRSLIQPGNIGYDGKAMYCRVHDTILDFLIDKSSEENMCTVLKKQCKPNGIVRRLSLMGNEDEEIVEQLDLSHARSITAFGDIKLLPSLGRSKCLRVLDLQDCDQLENHHIKDIERLYQLRYLDISSTGITELPRQIGELLYLETLV--AY-GLRELPE   640
PI330550_R6     481  VNRSLIQPGNIGYDGKAMYCRVHDTILDFLIDKSSEENMCTVLKKQCKPNGIVRRLSLMGNEDEEIVEQLDLSHARSITAFGDIKLLPSLGRSKCLRVLDLQDCDQLENHHIKDIERLYQLRYLDISSTGITELPRQIGELLYLETLV--AY-GLRELPE   640
PI306540_SrTm5  481  VNRSLIQPGNIGYDGKAMYCRVHDTILDFLIDKSSEENMCTVLKKQCKPNGIVRRLSLMGNEDEEIVEQLDLSHARSISAFGDIKLLPSLGRSKCLRVLDLQGCDQLENHHIKDIERLYQLRYLDISSTGITELPRQIGELLYLETLV--AY-GLRELPE   640
DV92_S1         481  VNRSLIQPGNIGYDGKAEYCRVHDTILDFLIDKSSEENMCTVLKKQCKPNGIVRRLSLMGNEDEEIMEQLDLSHARSISAFGDIKLLPSLGRSKCLRVLDLQGCYQLKNHHIKDIERLYQLRYLDISFTGITELPRQIGELLYLETLV-TSY-GLRELPE   640
IG44878_S2      481  VNRSLIQPGNIGYDGKAKYCRVHDTILDFLIDKSSEENMCTVLKKQCKPNGIVRRLSLMGNEDEEIMEQLDLSHARSISAFGDIKLLPSLGRSKCLRVLDLQGCYQLKNHHIKDIERLYQLRYLDISFTGITELPRQIGELLYLETLVSTSY-GLRELPE   640
PI355523_S3     481  VNRSLIQPGNIGYDGKAMYCRVHDTILDFLIDKSSEENMCTVLKKQCKPNGIVRRLSLMGNEDEEIVEQLDLSHARSISAFGDIKLLPSLGRSKCLRVLDLQDCDQLENHHIKDIERLYQLRYLDISYTGITELPRQIGELLYLETLD--AS-GLRELPE   640
PI573523_S4     481  VNRSLIQPGNIGYDGKAEYCRVHDTILDFLIDKSSEENMCTVLKKQCKPNGIVRRLSLMGNEDEEIMEQLDLSHARSISAFGDIKLLPSLGRSKCLRVLDLQGCYQLKNHHIKDIERLYQLRYLDISFTGITELPRQIGELLYLETLVSTSY-GLRELPE   640
WESTONIA_S5     481  VNRSLIQPDYIGYDGKTEYCRVHDTILDFLIDKSSEENMCTVLKKQCKPNGIVRRLSLMGNEDEEIVEQLDLSHARSISAFGDIKLLPSLGRSKCLRVLDLQNCGQLRNHHIKDIERLYQLRYLDISFTGITELPRQIGELLYLETLV--TSDGLRELPE   640
PI272557_S6     481  VNRSLIQPDNIGYDGKAKYCRVHDTILDFLIDKSSEENMCTVLKKQCKPNGIVRRLSLMGNEDEETVEQLDLSHARSITAFRDIKLLPSLGRSKCLRVLDLQACNQLENHHIKDIERLYQLRYLDISFTGITELPRQIGELLYLETLV--TSYRLRELPE   640

SCHOMBURGK_R1   641  STSRLQRLARLFVYSGCKLPGGLGNLINLQELDCVDALHLKHVEELGKLTNLRKLSIKLDTGGIEGNKLEESKEKLVSSLCKLDECGLLSLSIDYYLREKDGEEPFLPALGCIQEVFVYGQDISRISRWLASLPNLHRLLLDDP--KIEQQDIEMIGLI-   800
IG44855_R2      641  STSRLQRLARLFVYSGCKLPGGLGNLINLQELDCVDALHLKHVEELGKLTNLRKLSIKLDTGGIEGNKLEESKEKLVSSLCKLDECGLLSLSIDYYLREKDGEEPFLPALGCIQEVFVYGQDISRISRWLASLPNLHRLLLDDP--KIEQQDIEMIGLI-   800
IG44921_R3      641  STSRLQRLARLFVYSGCKLPGGLGNLINLQELDCVDALHLKHVEELGKLTNLRKLSIKLDTGGIEGNKLEESKEKLVSSLCKLDECGLLSLSIDYYLREKDGEEPFLPALGCIQEVFVYGQDISRISRWLASLPNLHRLLLDDP--KIEQQDIEMIGLI-   800
PI190945_R4     641  STSRLQRLARLFVYSGCKLPGGLGNLINLQELDCVDALHLKHVEELGKLTNLRKLSIKLDTGGIEGNKLEESKEKLVSSLCKLDECGLLSLSIDYYLREKDGEEPFLPALGCIQEVFVYGQDISRISRWLASLPNLHGLLLDNP--KIEQQDIEMIGLI-   800
PI289605_R5     641  STSRLQRLARLFVYSGCKLPGGLGNLINLQELDCVDALHLKHVEELGKLTNLRKLSIKLDTGGIEGNKLEESKEKLVSSLCKLDECGLLSLSIDYYLREKDGEEPFLPALGCIQEVFVYGQDISRISRWLASLPNLHGLLLDYP--KIEQQDIEMIGLI-   800
PI330550_R6     641  STSRLQRLARLFVYSGCKLPGGLGNLINLQELDCVDALHLKHVEELGKLTNLRKLSIKLDTGGIEGNKLEESKEKLVSSLCKLDECGLLSLSIDYYLREKDGEEPFLPALGCIQEVFVYGQDISRISRWLASLPNLHGLLLDNP--KIEQQDIEMIGLI-   800
PI306540_SrTm5  641  STSRLQRLARLFVYSGCKLPGGLGNLINLQELDCVDALHLKHVEELGKLTNLRKLSIKLDTGGIEGNKLEESKEKLVSSLCKLDECGLRSLSIHYYLREKDGEEPFLPALGCIQEVCVYGQDISRISRWLASLPNLHRLFLNQP--KIEQQDIEMIGLI-   800
DV92_S1         641  STSRLQRLARLFVYHGCKLPDGLGNLVNLQELECVDALQLKHVEELGKLTNLRKLSIKLDTGGIEGNKLEESKEKLVSSLCKLDECGLRSLSIHYYLREKDGEEPFLPALGCIQEVCVYGQDISRISRWLASLPNLHRLFFNDPKMKIEQQDIEMIGLI-   800
IG44878_S2      641  STSRLQRLARLFVDSGCKLPDGLGNLINLQELDCVDALQLKHVEELGKLTNLRKLRIKLDTDGIEGNKLEQSKEKLVSSLCKLDECGLLSLSIYYYLREKDGEEPFLPALGCIQEVFVHGQDISRISRWLASLPNLHRLFLNQP--KIEQQDIEMIALRE   800
PI355523_S3     641  STSRLQRLARLFVDSGCKLPDGLGNLINLQELDCVDALQLKHVEELGKLTNLRKLKIKLDTGGIEGNKLEESKEKLVSSLCKLDECGLRSLSIRYYLREKDGEEPFLPALGCIQEVCVYGQDISRISRWLASLPNLHTLLLDYP--KIEQQDIEMIGLI-   800
PI573523_S4     641  STSRLQRLARLFVDSGCKLPDGLGNLINLQELDCVDALQLKHVEELGKLTNLRKLRIKLDTDGIEGNKLEQSKEKLVSSLCKLDECGLLSLSIYYYLREKDGEEPFLPALGCIQEVFVHGQDISRISRWLASLPNLHRLFLNQP--KIEQQDIEMIGLI-   800
WESTONIA_S5     641  STSRLQRLARLIVGCDCKLPDGLGNLMNLQELDCVGALHLKHAEELGKLTNLRKLKINLYTHGIEGNKLEESKEKLVSSLCKLDECGLRSLSIDYYLREKDGEEPFLPALGCIEEVFVYEQDISRISRWLASLPNLHRLVSYDP--KIEQQDIEMIGLI-   800
PI272557_S6     641  STSRLQRLARLFVDPGCKLPDGLGNLINLQELDWVDALQLKHVEELGKLTNLRKLRIKLDTDGIEGNKLEQSKEKLVSSLCKLDECGLRSLSIGYYLREKDGEEPFLPPLGCIQEVSVYGQDISRISRWLASLPNLHMLFFDYV--KIEQQDIEMIGLI-   800

SCHOMBURGK_R1   801  PNLIDLTLPPLYKTDDA-GRLIIRREGFQQLQKFEAYNTRMGVLMFEPGAMPRLKELKLHNFIEKPKSAAVDFDFGIQRLSSLARLTVSLSCGGWTVAEVEAAEDAFKSMAEANPNRPILEMTRYNTQHMLQDEQIGMTGSATTPAVKS   949
IG44855_R2      801  PNLIDLTLPPLYKTDDA-GRLIIRREGFQQLQKFEAYNTRMGVLMFEPGAMPRLKELKLHNFIEKPKSAAVDFDFGIQRLSSLARLTVSLSCGGWTVAEVEAAEDAFKSMAEANPNRPILEMTRYNTQHMLQDEQIGMTGSATTPAVKS   949
IG44921_R3      801  PNLIDLTLPPLYKTDDA-GRLIIRREGFQQLQKFEAYNTRMGVLMFEPGAMPRLKELKLHNFIEKPKSAAVDFDFGIQRLSSLARLTVSLSCGGWTVAEVEAAEDAFKSMAEANPNRPILEMTRYNTQHMLQDEQIGMTGSATTPAVKS   949
PI190945_R4     801  PNLIDLTL-YLRITDDA-GRLIIKREGFQQLQRFELSRIRMGDLMFEPGAMPRLKELILYHFIEKPKSGAVDFDFGIQHLSSLARLTVGLLCVGSTAAEVEAAEDAFKSMAEANPNRPILEMTRLYPQHMLQDEQIGMTGSATTPAVKS   949
PI289605_R5     801  PNLIDLTL-YLRITDDA-GRLIIKREGFQQLQRFELSRIRMGDLMFEPGAMPRLKELILYHFIEKPKSGAVDFDFGIQHLSSLARLTVGLLCVGSTAAEVEAAEDAFKSMAEANPNRPILEMTRLYPQHMLQDEQIGMTGSATTPAVKS   949
PI330550_R6     801  PNLIDLTL-YLRITDDA-GRLIIKREGFQQLQRFELSRIRMGDLMFEPGAMPRLKELILYHFIEKPKSGAVDFDFGIQHLSSLARLTVGLLCVGSTAAEVEAAEDAFKSMAEANPNRPILEMTRLYPQHMLQDEQIGMTGSATTPAVKS   949
PI306540_SrTm5  801  PNLIDLSL-SLYKTDDA-GRLIIRREGFQQLQSFRVYHTRMGVLMFEPGAMPRLKELKFYDFIGKPKSGAVDFDFGIQHLSSLARLTVDLSCVGWTAAEVEAAEDAFKSMAEANPNRPILEMTRLFPQDMLRDEQIDMAGSATTPAVKS   949
DV92_S1         801  PNLMDLTL-YLRITDDAMPRLIIKREGFQQLQRFELSRIRMGDLMFEPGAMPRLKELILHNFIEKPKSGAVDFDFGIQHLSSLARLTVGLLCVGSTAAEVEAAEDAFKSMAEANPNRPILEMTRLCPQHMLQDEQIGMTGSATTPAVKS   949
IG44878_S2      801  PNLMDLTL-YLCITDDA-GRLIIKREGFQQLQRFELSRIRMGDLMFEPGAMPRLKELILYDFIGKPKSGAVDFDFGIQHLSSLARLTVGLSCVGSTVAEVEAAEDAFKSMAEANPNRPILEMTRYNTQHMLQDEQIGMTGSATTPAVKS   949
PI355523_S3     801  PNLIDLTL-PLYKTDDA-GRLIIRREGFQQLQKFEAYNTRMGVLMFEPGVMPRLKELKLHNFIEKPKSAAVDFDFGIQHLSSLARLTVDLSCVGWTAAEVEAAEDAFKSMAEANPNRPILEMTRYNTQHMLQDEQIGMTGSATTPAVKS   949
PI573523_S4     801  PNLMDLTL-YLCITDDA-GRLIIKREGFQQLQRFELSRIRMGDLMFEPGAMPRLKELILYDFIGKPKSGAVDFDFGIQHLSSLARLTVGLSCVGSTVAEVEAAEDAFKSMAEANPNRPILEMTRYNTQHMLQDEQIGMTGSATTPAVKS   949
WESTONIA_S5     801  PNLIDLTL-SLPGTDDA-GRFIITREGFQQLQSFELSGSRMGVL-FEPGAMPRLKELILDDFIRKPKSAAVDFDFGIQRLSSLARLTVSLACYRSTAAEVEATEDAFKSMAEANPNRPILEMTRLYPQHMVRDEQIDMAGSATTPAVKS   949
PI272557_S6     801  PNLIDLSL-SLRKTDDA-GRLIIRREGFQHLHSFRVYDTRMGVLMFEPGAMPRLKELKLHNFIEKPESGAVDFDFGIQRLSSLARLTVSLFCVGSTAAEVEATEDAFKSMAEANPNRPILEMIRVNPHRMF-DEQIDMAGSATTPAVKS   949
```

**Figure S3** SrTm5 protein sequence analysis. Multiple sequence alignment between SrTm5 and reported Sr22 resistant and susceptible protein sequences (Steuernagel et al. 2016). Highlighted in green are protein polymorphisms that discriminate perfectly between Sr22 susceptible and resistant haplotypes. Highlighted in yellow are Sr22b unique protein polymorphisms compared with other Sr22 resistant haplotypes.

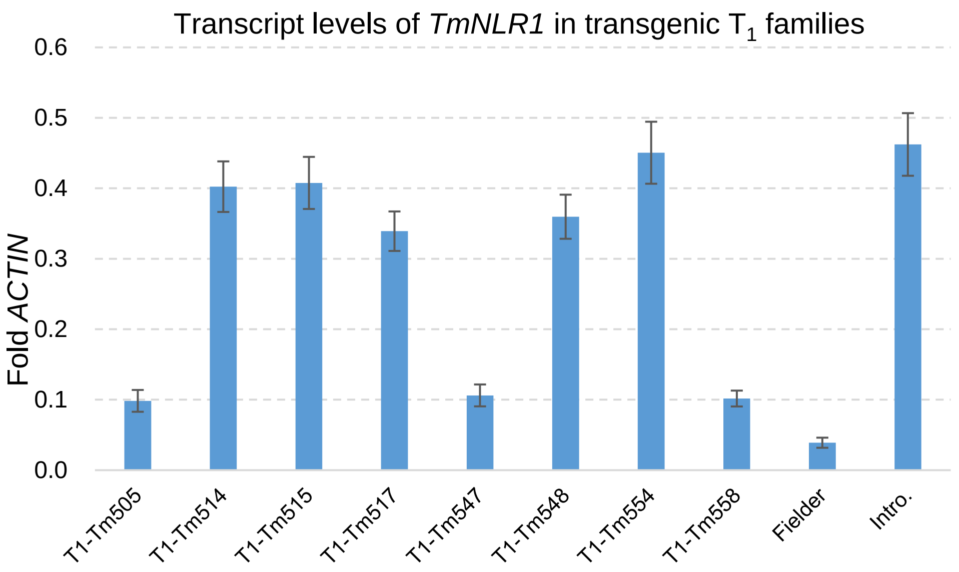


**Figure S4** Transcript levels of *TmNLR1* in transgenic $T_1$ families (three positive plants per event, n = 3). Transcript levels are expressed as fold-*ACTIN* using the $2^{\Delta CT}$ method. Fielder, susceptible control; Intro., *SrTm5* introgression plants ($BC_3F_2$, positive control). Error bars are standard errors of the mean.

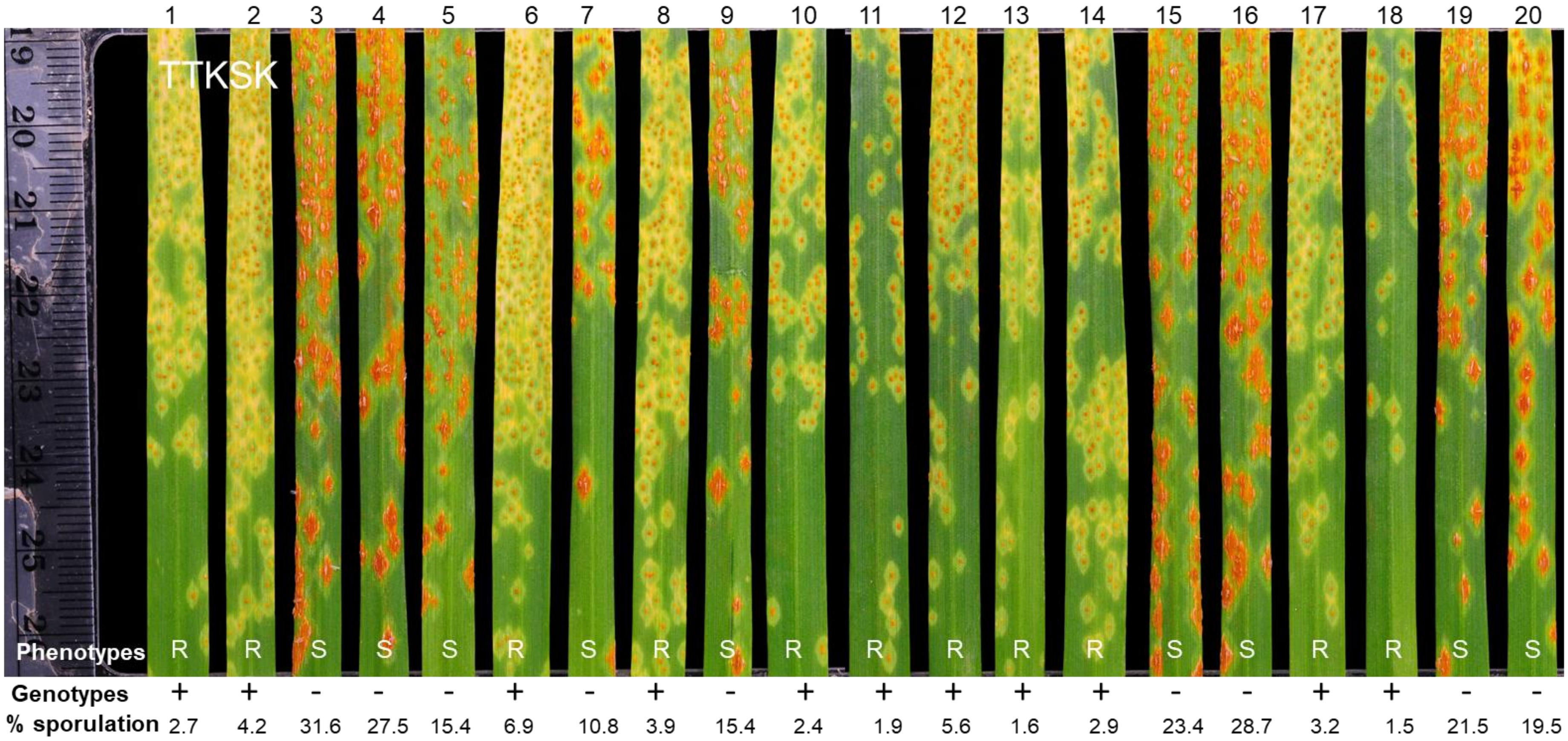


**Figure S5** Reactions to *Pgt* race TTKSK (Ug99) in transgenic family $T_2$Tm515-6. This family showed segregation for resistance. Genotyping of the plants with markers *Tm5F3R4*, *TM5TF2R2*, and *TM5TF3R3* (Table 1) revealed a perfect co-segregation between the presence of transgene and the phenotypes. The numbers below the figure indicate the average percentage of the leaf area covered by *Pgt* pustules. S, susceptible; R, resistant; +, with transgene; -, without transgene.

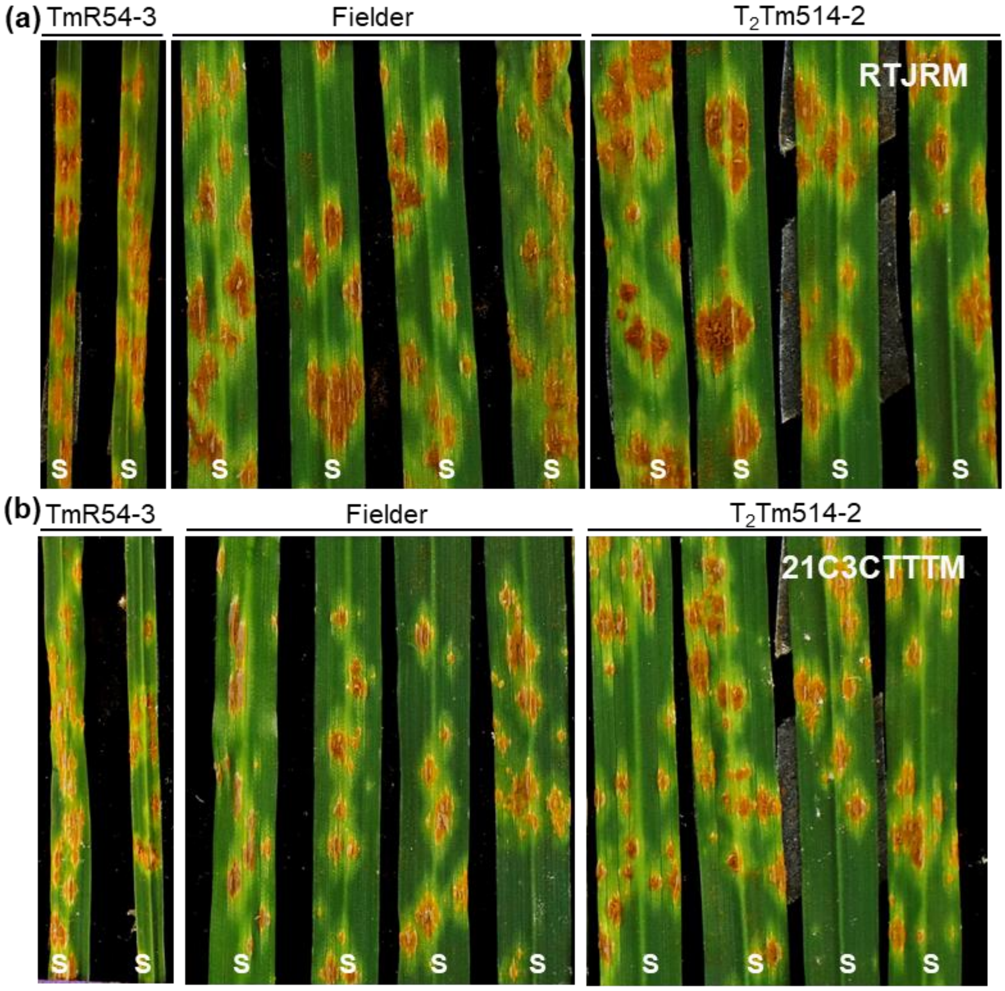


**Figure S6** Transgenic family $T_2$Tm514-2 homozygous for the transgene were inoculated with two *SrTm5*-virulent *Pgt* races RTJRM and 21C3CTTTM. (a) Stem rust reactions to *Pgt* race RTJRM. (b) Stem rust reactions to *Pgt* race 21C3CTTTM. All the tested plants from transgenic family $T_2$Tm514-2 were susceptible to these two races suggesting similar race specificity between the transgene and natural *SrTm5* in *T. monococcum*. Figure 2a in the main text presents stem rust reactions from the same transgenic family $T_2$Tm514-2 against *Pgt* race TTKSK.

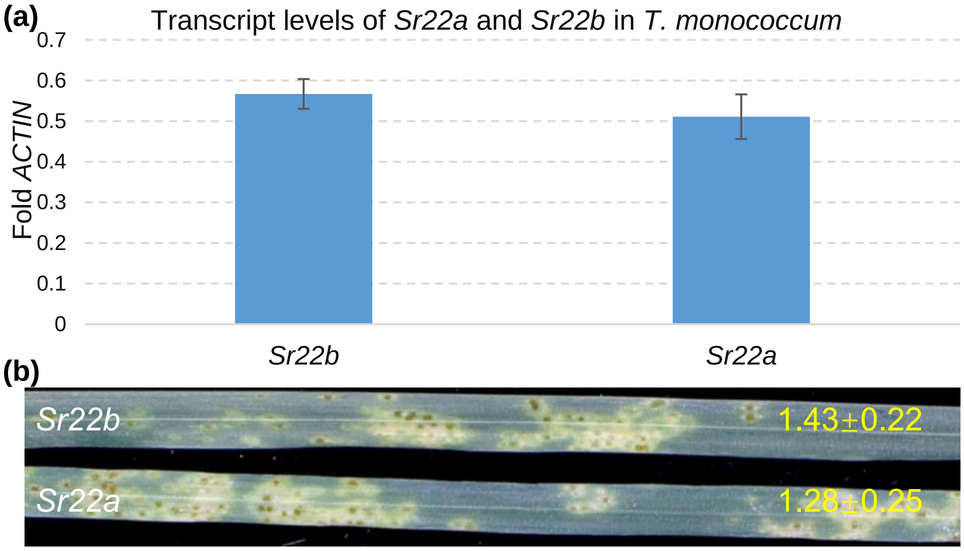


**Figure S7** Transcript levels and infection types of *Sr22a* and *Sr22b* in *T. monococcum* background. (a) Transcript levels of *Sr22a* and *Sr22b* in *T. monococcum*. Leaves were collected from *T. monococcum* accession PI 190945 (*Sr22a*) and *T. monococcum* line TmR54-3 (*Sr22b*) without *Pgt* inoculation. Plants were grown in growth chambers at 22 ˚C day / 20 ˚C night with 16 hours light / 8 hours dark. Transcript levels were expressed as fold-*ACTIN* (n = 6). Error bars are standard errors of the mean. (b) Infection types on *T. monococcum* plants of PI 190945 (*Sr22a*) and TmR54-3 (*Sr22b*) when inoculated with *Pgt* race 34PKUSC. No significant differences were observed between the two genotypes in average percentage of leaf area covered by *Pgt* pustules using software ASSESS v2 (n = 5).

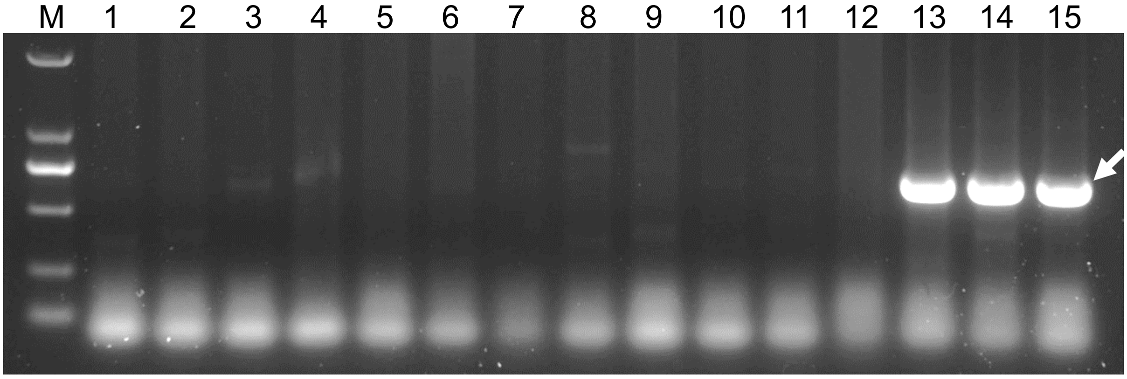


**Figure S8** PCR products from the *Sr22b* diagnostic marker *TM5TF2R2*. The amplicons of 673 bp (white arrow) were present only in resistant *T. monococcum* accessions carrying *Sr22b*. 1, Xiaoyan22 (6x); 2, Chinese Spring (6x); 3, Chuanmai42 (6x); 4, Fielder (6x); 5, 8155B (4x); 6, Rusty (4x); 7, D447 (4x); 8, Kronos (4x); 9, PI 272557 (2x); 10, PI 418580 (2x); 11, PI 487249 (2x); 12, PI 427507 (2x); 13, PI 306540 (2x); 14, PI 377668 (2x); 15, PI 362610 (2x); M, markers.

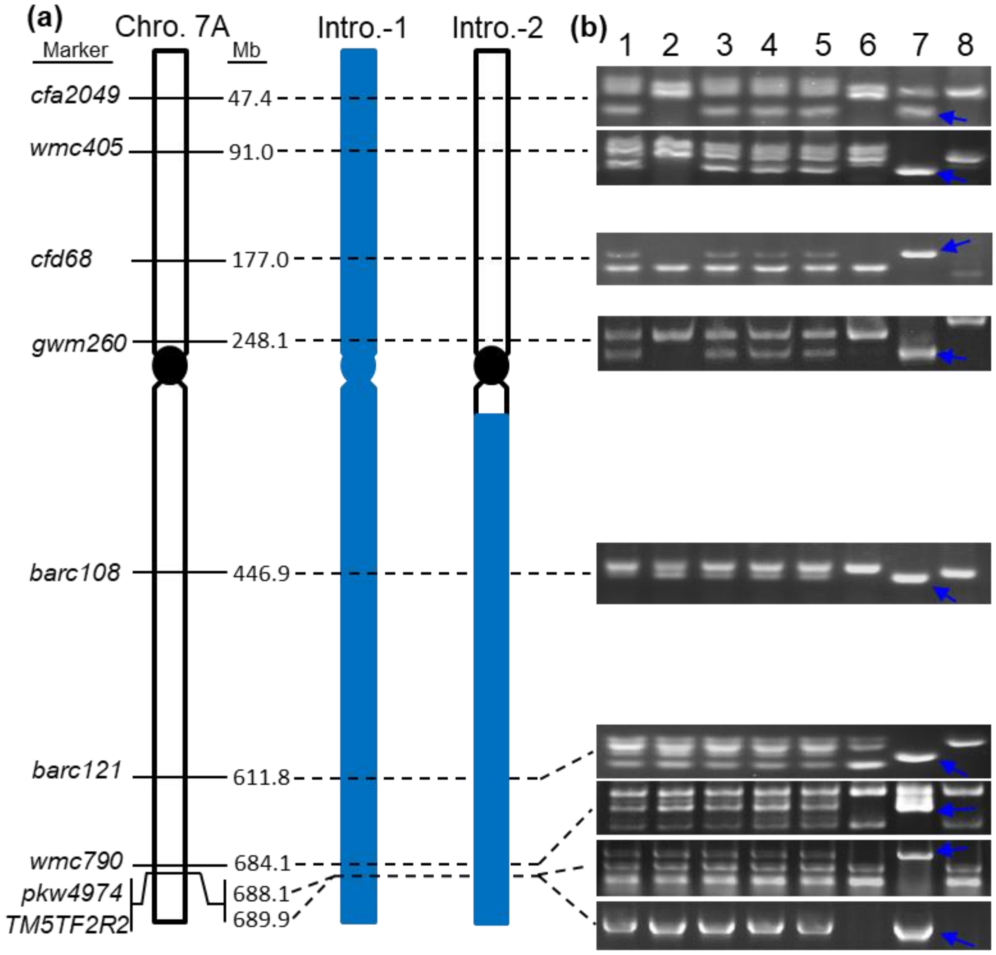


**Figure S9** Markers across chromosome 7A were used to analyze the length of introgressed *T. monococcum* segments. (a) The physical positions of markers and the length of the introgression segments. Blue rectangles indicate *T. monococcum* chromatin. The positions were based on the reference genome of Chinese Spring Refseq v1.0. (b) PCR products from nine selected markers. 1-5, $BC_3F_1$ plants 1, 2, 3, 4 and 5; 6, Fielder; 7, PI 306540; and 8, Kronos. Blue arrows indicate the bands from PI 306540.

**Table S1.** Avirulence/virulence formulae of *Pgt* races, and their responses to *SrTm5*. The data shown here was based on the current study and several previous studies (Li et al. 2016; Chao et al. 2017; Li et al. 2018; Chen et al. 2018a; Chen et al. 2020).

| **Race (isolate)**[#] | **Origin** | **Response to *SrTm5*** | **Avirulence** | **Virulence** |
|---|---|---|---|---|
| BCCBC (09CA115-2) | USA | virulence | *Sr5 6 7b 8a 9a 9d 9e 9b 10 11 21 24 30 31 36 38 Tmp* | *Sr9g 17 McN* |
| TTKSK (04KEN156/04) | Kenya | **avirulence** | *Sr21 24 36 Tmp* | *Sr5 6 7b 8a 9a 9b 9d 9e 9g 10 11 17 30 31 38 McN* |
| MCCFC (59KS19) | USA | **avirulence** | *6 8a 9a 9d 9e 9b 11 21 24 31 30 36 38* | *5 7b 9g 10 17 McN Tmp* |
| TTKST (06KEN19-V-3) | Kenya | **avirulence** | *21 36 Tmp* | *5 6 7b 8a 9a 9b 9d 9e 9g 10 11 17 24 30 31 38 McN* |
| 34MTGSM (20GSA1) | China | **avirulence** | *Sr9e 12 13 14 17 21 22 23 26 30 31 33 35 36 37 38 47 Tmp* | *Sr5 6 7b 8a 9a 9b 9d 9f 9g 10 11 15 16 18 19 20 24 25 27 28 29 32 34 39 McN* |
| QFCSC (06ND76C) | USA | virulence | *6 7b 9e 9b 11 24 31 30 36 38 Tmp* | *5 8a 9a 9d 9g 10 17 21 McN* |
| TRTTF (06YEM34-1) | Yemen | virulence | *8a 24 31* | *5 6 7b 9a 9b 9d 9e 9g 10 11 17 21 30 36 38 McN Tmp* |
| TTTTF (01MN84A-1-2) | USA | virulence | *24 31* | *5 6 7b 8a 9a 9b 9d 9e 9g 10 11 17 21 30 36 38 McN Tmp* |
| 21C3CTTTM (20GH13) | China | virulence | *Sr5 9e 14 19 21 22 23 26 27 31 33 35 37 38 39 47* | *Sr6 7b 8a 9a 9b 9d 9f 9g 10 11 12 13 15 16 17 18 20 24 25 28 29 30 32 34 36 Tmp McN* |
| RTJRM (mutant strain, 20IAS11) | China | virulence | *Sr13 14 17 18 19 20 22 23 25 27 28 29 31 32 33 36 37* | *Sr5 8a 10 11 12 15 21 24 30 34 35 Tmp McN* |

[#], Chinese race 34PKUSC (19IAS08) was provided by Dr. Jianhui Wu at Northwest Agriculture & Forestry University in China, and its responses to different *Sr* resistance genes is unknown.

**Table S2.** Comparison of SrTm5 protein with polymorphisms that discriminate perfectly between Sr22 susceptible and resistant haplotypes from Steuernagel et al. (2016). SrTm5 carries the same amino acids as the resistant haplotypes at all three positions 381, 605 and 655. BLOSUM62 scores were obtained from the following link (https://www.ncbi.nlm.nih.gov/IEB/ToolBox/C_DOC/lxr/source/data/BLOSUM62). The resistant (PI 306540) and susceptible (PI 272557) parents are indicated in red.

| Positions | Accessions | | | | | | | | | | | | | BLOSUM62 |
|---|---|---|---|---|---|---|---|---|---|---|---|---|---|---|
| | SCHOMBURGK<br>R1 | IG44855<br>R2 | IG44921<br>R3 | PI190945<br>R4 | PI289605<br>R5 | PI330550<br>R6 | **PI306540**<br>***SrTm5*** | DV92<br>S1 | IG44878<br>S2 | PI355523<br>S3 | PI573523<br>S4 | Westonia<br>S5 | **PI272557**<br>**S6** | |
| **381** | V | V | V | V | V | V | **V** | L | L | L | L | L | **L** | **1** |
| **605** | S | S | S | S | S | S | **S** | F | F | Y | F | F | **F** | **-2** |
| **655** | G | G | G | G | G | G | **G** | D | D | D | D | D | **D** | **-1** |
| Phenotypes | Res. | Res. | Res. | Res. | Res. | Res. | **Res.** | Sus. | Sus. | Sus. | Sus. | Sus. | **Sus.** | |

**Table S3.** Segregation ratios in $T_1$ and $T_2$ transgenic families detected using PCR markers *Tm5F3R4*, *TM5TF2R2*, and *TM5TF3R3* (Table 1). We genotyped more than 20 $T_1$ plants from each transgenic family. All transgenic $T_1$ families except $T_1$-Tm505 showed significant segregation distortion with an excess of non-transgenic plants. Genotyping of $T_2$ plants from one positive $T_1$ plant for each transgenic event revealed that the transgene was fixed in families $T_2$-Tm505-15, $T_2$-Tm514-2, $T_2$-Tm517-1, $T_2$-Tm548-3, $T_2$-Tm554-2 and $T_2$-Tm558-7, and segregating in families $T_2$-Tm515-6 and $T_2$-Tm547-3.

| Genotyping | | Transgenic $T_1$ families | | | | | | | |
|---|---|---|---|---|---|---|---|---|---|
| | | $T_1$-Tm505 | $T_1$-Tm514 | $T_1$-Tm515 | $T_1$-Tm517 | $T_1$-Tm547 | $T_1$-Tm548 | $T_1$-Tm554 | $T_1$-Tm558 |
| Present (+) | | 22 | 8 | 6 | 10 | 15 | 12 | 8 | 3 |
| Absent (-) | | 3 | 20 | 16 | 88 | 45 | 20 | 20 | 18 |
| $\chi^2$ *P* value | 1 copy | 0.133 | 1.4E-08 | 2.3E-07 | 1.2E-49 | 3.7E-19 | 9.6E-07 | 1.4E-08 | 1.3E-10 |
| Genotyping | | Transgenic $T_2$ families | | | | | | | |
| | | $T_2$-Tm505-15 | $T_2$-Tm514-2 | $T_2$-Tm515-6 | $T_2$-Tm517-1 | $T_2$-Tm547-3 | $T_2$-Tm548-3 | $T_2$-Tm554-2 | $T_2$-Tm558-7 |
| Present (+) | | 23 | 33 | 23 | 39 | 12 | 22 | 24 | 21 |
| Absent (-) | | 0 | 0 | 16 | 0 | 12 | 0 | 0 | 0 |
| $\chi^2$ *P* value | 1 copy | 0.006 | 9.1E-04 | 0.021 | 3.1E-04 | 0.005 | 0.007 | 0.005 | 0.008 |

**Table S4.** Resistance profiles of *Sr22b* (=*SrTm5*) and *Sr22a* (haplotypes R1 and R4) to multiple *Pgt* races. Infection types for *Sr22b* are based on the selected $F_5$ line TmR54-3, whereas *Sr22a* infection types are from *T. aestivum* lines Schomburgk / PI 660256 (R1) and *T. monococcum* accession PI 190945 (R4). PI 272557 was used as susceptible control.

| *Pgt* race | *Sr22b* 2x[a] TmR54-3 | *Sr22a* (*Sr22TB*, R1) 6x[a] Schomburgk/PI 660256 | *Sr22a* (R4) 2x[a] PI 190945 | No *Sr* gene 2x[a] PI 272557 |
|---|---|---|---|---|
| TTKSK[b] | ;1 | 2- | 1 | 4 |
| MCCFC[b] | 1; | 2- | ;1 | 4 |
| TTKST | ; | Not available | Not available | Not available |
| 34PKUSC | 1 | 2- | ;1 | 3+ |
| 34MTGSM | 1; | 2- | ;1 | 3+ |
| BCCBC | 3+ | 2- | ;1 | 3+ |
| QFCSC[b] | 3+ | ;2- | ;1- | 4 |
| TRTTF[b] | 3+ | 2-; | 1; | 3+ |
| TTTTF[b] | 3+ | 2 | 2- | 4 |

[a], 2x = diploid wheat (*T. monococcum*), 6x= hexaploid wheat (*T. aestivum*).

[b], Based on previous studies (Steuernagel et al. 2016, Rouse & Jin, 2011 and Chen et al. 2018a)

**Table S5.** A collection of 92 accessions of *T. monococcum*, 23 of *T. turgidum*, and 53 of *T. aestivum* was used to test the presence of *Sr22b*. All these accessions were screened using the diagnostic marker *TM5TF2R2* and the other two sequence-based markers *TM5AF6R8* and *TM5AF4R4*. PI, CItr and GSTR numbers correspond to Germplasm Resources Information Network (GRIN) numbers.

| **Accessions** | **Acc. No.** | **With/without *Sr22b*** |
|---|---|---|
| ***T. monococcum***: PI 306540, PI 277130, PI 306545, CItr 17657, PI 277131-2, PI 306544, PI 306547, PI 352480, PI 355536, PI 355541, PI 435000, PI 435001, PI 221414, **PI 355538, PI 362610, PI 377668** | 16 | with (+) |
| ***T. monococcum***: PI 418580, PI 487249, CItr 17671, PI 427507, PI 272560, PI 427580, CItr 17674, PI 245726, PI 272557, PI 573520, PI 560720, G3116, DV92, PI 573523, PI 427464, PI 427444, PI 352273, PI 427465, PI 272556, CI2433, PI 427498, PI 352505, PI 190942, PI 277121, CItr 14520, CItr 13963, PI 427662, PI 427478, CItr 13964, CItr 17655, PI 168803, PI 168806, PI 190940, PI 289605, PI 352486, PI 355517, PI 355524, PI 428158, PI 503874, PI 427452, PI 330528, PI 554480, PI 427467, PI 428012, PI 538540, PI 362554, PI 427497, PI 427993, PI 427476, CItr 17741, PI 427450, PI 554519, PI 427661, PI 427405, PI 427540, PI 352504, PI 427453, PI 427510, PI 427603, CItr 17665, PI 355453, PI 427477, PI 427484, PI 427488, PI 554517, PI 401412, PI 427994, PI 427472, PI 427468, PI 352270, PI 427592, PI 427796, PI 427527, PI 427808, PI 190945, PI 362553 | 76 | without (-) |
| ***T. turgidum***: 8155B, Rusty, D447, Kronos, Svevo, LineE, CItr 15326, PI 478298, PI 584833, CItr 13768, PI 331260, PI 480016, CItr 15892, PI 510696, Zavitan, PI 94701, PI 606286, PI 480148, PI 191365, CItr 13165, PI 496260, PI 560877, PI 428016 | 23 | without (-) |
| ***T. aestivum***: Xiaoyan22, Zhengzhou5389, Chinese Spring, Cadenza, Lassik, PI 675640, Patwin, UC1110, PI 660056, ISr8a-Ra, CItr 15082, PI 660060, PI 178759, PI 648419, PI 679621, PI 181434, PI 660059, PI 182527, PI 189747, PI 430067, PI 660057, PI 648417, PI 442904, PI 603918, PI 600683, GSTR434, GSTR429, GSTR428, GSTR420, GSTR501, GSTR522, GSTR425, PI 679605, GSTR409, PI 679598, PI 679603, GSTR437, PI 566596, GSTR441, Avocet-S, Pavon, Fielder, PI 638738, Taichang29, SY95-71, PI 675564, PI 672538, PI 277012, PI 596533, Chuanmai42, Xinong511, Taimai198, PI 634936 | 53 | without (-) |

**Table S6.** Geographic distribution of *T. monococcum* accessions, and their reactions against *Pgt* races TTKSK, MCCFC and 34PKUSC. The data for races TTKSK and MCCFC was based on the previous study (Rouse & Jin, 2011). Gene postulations were based upon infection types and genotypes from the diagnostic markers of cloned *Sr* genes *Sr21*, *Sr22*, *Sr35* and *Sr60* (Saintenac et al., 2013, Steuernagel et al., 2016, Chen et al., 2018b, Chen et al., 2020). +, with *Sr22b*; -, without *Sr22b*.

| Accession | With / without *Sr22b* | Source | Species[a] | TTKSK | MCCFC | 34PKUSC | Gene Postulation |
|---|---|---|---|---|---|---|---|
| TmR54-3 | (+) | - | *T. m. m* | ;1 | **1;** | **1** | ***Sr22b*** |
| PI 306540 | (+) | Romania | *T. m. m* | **0;** | **0** | **0** | *Sr21, SrTm4, Sr60,* ***Sr22b*** |
| PI277131-2 | (+) | Albania | *T. m. m* | **;** | **;1/;** | **;1** | *Sr21, Sr60,* ***Sr22b*** |
| PI 277130 | (+) | Albania | *T. m. m* | **;** | **0;** | **;1** | *Sr21, Sr60,* ***Sr22b*** |
| PI 306545 | (+) | Romania | *T. m. m* | **;1-** | **0;** | **;1** | *Sr21, Sr60,* ***Sr22b*** |
| CItr 17657 | (+) | United States | *T. m. m* | **;** | **0;** | **;1** | *Sr21,* ***Sr22b*** |
| PI 306544 | (+) | Romania | *T. m. m* | **0;** | **0;** | **;1** | *Sr21, Sr60,* ***Sr22b*** |
| PI 306547 | (+) | Romania | *T. m. m* | **;** | **0;** | **;1** | *Sr21, Sr60,* ***Sr22b*** |
| PI 352480 | (+) | Albania | *T. m. m* | **0;** | **0;** | **;1-** | *Sr21, Sr60,* ***Sr22b*** |
| PI 355536 | (+) | Italy | *T. m. m* | **0** | **;1** | **0** | *Sr21, Sr35,* ***Sr22b*** |
| PI 355541 | (+) | Albania | *T. m. m* | **0;** | **0;** | **;1-** | *Sr21, Sr60,* ***Sr22b*** |
| PI 435000 | (+) | Yugoslavia | *T. m. m* | **0;** | **;** | **;1-** | *Sr21, Sr60,* ***Sr22b*** |
| PI 435001 | (+) | Bosnia and Herzego. | *T. m. m* | **;** | **;** | **0;** | *Sr21, Sr60,* ***Sr22b*** |
| PI 221414 | (+) | Yugoslavia | *T. m. m* | **0** | **0;** | **;1-** | *Sr21, Sr60,* ***Sr22b*** |
| PI 355538 | (+) | Balkans | *T. m. m* | **;1-** | **;1-** | **1** | ***Sr22b*** |
| PI 377668 | (+) | Former Yugoslavia | *T. m. m* | **;** | **0;** | **1** | ***Sr22b*** |
| PI 362610 | (+) | Macedonia | *T. m. m* | **;1-** | **0;** | **1** | ***Sr22b*** |
| PI 418580 | (-) | Azerbaijan | *T. m. a.* | 3+ | 4 | - | *Susceptible* |
| PI 487249 | (-) | Syria | *T. m. a.* | 2+/3/12+Z | ;1 | - | *Sr21* |
| CItr 17671 | (-) | Turkey | *T. m. a.* | 1/12Z | ;1 | - | *Sr21* |
| PI 427507 | (-) | Turkey | *T. m. a.* | 3- | 1 | - | *Sr21* |
| PI 272560 | (-) | Hungary | *T. m. m* | 0 | 4 | - | *Sr35* |
| PI 427580 | (-) | Turkey | *T. m. a.* | 123Z | ;1 | - | *Sr21* |
| CItr 17674 | (-) | Iran | *T. m. a.* | 2++ | 1 | - | *Sr21* |
| PI 245726 | (-) | Turkey | *T. m. a.* | - | 4 | - | *Susceptible* |
| PI 272557 | (-) | Hungary | *T. m. m* | 4 | 4 | 4 | *Susceptible* |
| PI 573520 | (-) | Turkey | *T. m. a.* | 2- | ;1 | - | *Sr22* |
| PI 560720 | (-) | Turkey | *T. m. m* | 2- | 4 | - | *Susceptible* |
| G3116 | (-) | Lebanon | *T. m. a.* | 2,2+ | 1 | - | *Sr21* |
| DV92 | (-) | - | *T. m. m* | 0; | 1 | - | *Sr21, Sr35* |
| PI 573523 | (-) | Turkey | *T. m. m* | ;N | ;1- | - | *Sr22* |
| PI 427464 | (-) | Azerbaijan | *T. m. a.* | 2+3Z | ;1 | - | *Sr21* |

| | | | | | | | |
|---|---|---|---|---|---|---|---|
| PI 427444 | (-) | Turkey | *T. m. a.* | 22+Z | 1 | - | *Sr21* |
| PI 352273 | (-) | Asia Minor | *T. m. a.* | 12Z | 1 | - | *Sr21* |
| PI 427465 | (-) | Armenia | *T. m. a.* | 2+3Z | 2 | - | *Sr21* |
| PI 272556 | (-) | Hungary | *T. m. a.* | 4 | 4 | 4 | *Susceptible* |
| CI 2433 | (-) | Germany | *T. m. m* | 2+ | ;1 | - | *Sr21* |
| PI 427498 | (-) | Turkey | *T. m. a.* | 3 | 22+ | - | *Sr21* |
| PI 352505 | (-) | Switzerland | *T. m. a.* | ;1 | ;1 | - | *Sr22* |
| PI 190942 | (-) | Spain | *T. m. m* | 1 | ;1 | - | *Sr21* |
| PI 277121 | (-) | Germany | *T. m. a.* | 2+3Z | ;1 | - | *Sr21* |
| CItr 14520 | (-) | Canada | *T. m. m* | 2 | ;1 | - | *Sr21* |
| CItr 13963 | (-) | United States | *T. m. m* | 2+ | ;1 | - | *Sr21* |
| PI 427662 | (-) | Iraq | *T. m. a.* | 3+ | 33+ | - | *Susceptible* |
| PI 427478 | (-) | Turkey | *T. m. a.* | 1/12Z | ;1 | - | *Sr21* |
| CItr 13964 | (-) | United States | *T. m. m* | 3- | ;1 | - | *Sr21* |
| CItr 17655 | (-) | United States | *T. m. m* | 3- | ;1 | - | *Sr21* |
| PI 168803 | (-) | United States | *T. m. m* | 3- | ;1 | - | *Sr21* |
| PI 168806 | (-) | United States | *T. m. m* | 22+3Z | ;1 | - | *Sr21* |
| PI 190940 | (-) | Spain | *T. m. m* | 3- | ;1 | - | *Sr21* |
| PI 289605 | (-) | United Kingdom | *T. m. m* | 2- | ;1 | - | *Sr22* |
| PI 352486 | (-) | Switzerland | *T. m. m* | 2- | ;1 | - | *Sr22* |
| PI 355517 | (-) | Asia Minor | *T. m. m* | 12- | ;1 | - | *Sr22* |
| PI 355524 | (-) | Germany | *T. m. m* | ;1 | ;1 | - | *Sr22* |
| PI 428158 | (-) | United Kingdom | *T. m. m* | ;12- | ;1 | - | *Sr21, Sr60* |
| PI 503874 | (-) | South Africa | *T. m. m* | 3+ | ;1 | - | *Susceptible* |
| PI 427452 | (-) | Turkey | *T. m. a.* | 12Z | 1 | - | *Sr21* |
| PI 330528 | (-) | United Kingdom | *T. m. a.* | 1; | ;1 | - | *Sr22* |
| PI 554480 | (-) | Turkey | *T. m. a.* | 3- | ;1 | - | *Sr21* |
| PI 427467 | (-) | Azerbaijan | *T. m. a.* | 2+3Z | 2 | - | *Sr21* |
| PI 428012 | (-) | Armenia | *T. m. a.* | 12+Z | ;1 | - | *Sr21* |
| PI 538540 | (-) | Turkey | *T. m. a.* | 33+ | 4 | - | *Susceptible* |
| PI 362554 | (-) | Yugoslavia | *T. m. m* | ;1 | ;1 | - | *Sr22* |
| PI 427497 | (-) | Turkey | *T. m. a.* | 22+Z | 1 | - | *Sr21* |
| PI 427993 | (-) | Lebanon | *T. m. a.* | 123Z/1;/12Z | ;1 | - | *Sr21* |
| PI 427476 | (-) | Turkey | *T. m. a.* | 1 | ;1 | - | *Sr21* |
| CItr 17741 | (-) | United States | *T. m. a.* | 2++ | ;1 | - | *Sr21* |
| PI 427450 | (-) | Turkey | *T. m. a.* | 3- | ;1 | - | *Sr21* |
| PI 554519 | (-) | Former Soviet Union | *T. m. a.* | 3+ | 4 | - | *Susceptible* |
| PI 427661 | (-) | Iraq | *T. m. a.* | 3 | 3+ | - | *Susceptible* |
| PI 427405 | (-) | Iraq | *T. m. a.* | 2+3Z | 1 | - | *Sr21* |
| PI 427540 | (-) | Turkey | *T. m. a.* | 2+3Z | 1 | - | *Sr21* |
| PI 352504 | (-) | Germany | *T. m. a.* | 2- | 1 | - | *Sr22* |
| PI 427453 | (-) | Turkey | *T. m. a.* | 22+Z | 1 | - | *Sr21* |
| PI 427510 | (-) | Turkey | *T. m. a.* | 3 | 22+ | - | *Sr21* |
| PI 427603 | (-) | Turkey | *T. m. a.* | 2+3Z | ;1 | - | *Sr21* |

| | | | | | | | |
|---|---|---|---|---|---|---|---|
| Cltr 17665 | (-) | Iran | *T. m. a.* | 1; | 1 | - | *Sr21* |
| PI 355453 | (-) | Asia Minor | *T. m. a.* | 2 | 1 | - | *Sr21* |
| PI 427477 | (-) | Turkey | *T. m. a.* | 12-Z | ;1 | - | *Sr21* |
| PI 427484 | (-) | Turkey | *T. m. a.* | 2/2+Z | ;1 | - | *Sr21* |
| PI 427488 | (-) | Turkey | *T. m. a.* | 22+Z | 22+ | - | *Sr21* |
| PI 554517 | (-) | Former Soviet Union | *T. m. a.* | 1 | ;1 | - | *Sr22* |
| PI 401412 | (-) | Iran | *T. m. a.* | 3 | 3+ | - | *Susceptible* |
| PI 427994 | (-) | Lebanon | *T. m. a.* | 12+Z | ;1+ | - | *Sr21* |
| PI 427472 | (-) | Turkey | *T. m. a.* | 1 | ;1 | - | *Sr21* |
| PI 427468 | (-) | Azerbaijan | *T. m. a.* | 2+3Z | 1 | - | *Sr21* |
| PI 352270 | (-) | Germany | *T. m. a.* | 2+3Z | 1 | - | *Sr21* |
| PI 427592 | (-) | Turkey | *T. m. a.* | 12+Z | ;1 | - | *Sr21* |
| PI 427796 | (-) | Iran | *T. m. a.* | 1 | ;1 | - | *Sr21* |
| PI 427527 | (-) | Turkey | *T. m. a.* | 12+Z | ;1 | - | *Sr21* |
| PI 427808 | (-) | Iran | *T. m. a.* | 1 | ;1 | - | *Sr21* |
| PI 190945 | (-) | Spain | *T. m. m* | 1 | ;1 | - | *Sr22* |
| PI 362553 | (-) | Yugoslavia | *T. m. m* | 1 | ;1 | - | *Sr22* |

[a], *T. m. m.* = *Triticum monococcum* subsp. *monococcum*; *T. m. a.* = *Triticum monococcum* subsp. *aegilopoides*.

## References


**Li, T., Wu, X., Xu, X., Wang, W. and Cao, Y.** (2016) Postulation of seedling stem rust resistance genes of Yunnan wheat cultivars in China. *Plant Protect Sci*, **52**, 242-249.

**Chao, S., Rouse, M.N., Acevedo, M., Szabo‐Hever, A., Bockelman, H., Bonman, J.M., Elias, E., Klindworth, D. and Xu, S.** (2017) Evaluation of genetic diversity and host resistance to stem rust in USDA NSGC durum wheat accessions. *The plant genome*, https://doi.org/10.3835/plantgenome2016.07.0071.

**Li, T.Y., Ma, Y.C., Wu, X.X., Chen, S., Xu, X.F., Wang, H., Cao, Y.Y. and Xuan, Y.H.** (2018) Race and virulence characterization of *Puccinia graminis* f. sp. *tritici* in China. *Plos One*, **13**, e0197579.

**Chen, S., Guo, Y., Briggs, J., Dubach, F., Chao, S., Zhang, W., Rouse, M.N. and Dubcovsky, J.** (2018a) Mapping and characterization of wheat stem rust resistance genes *SrTm5* and *Sr60* from *Triticum monococcum*. *Theor Appl Genet*, **131**, 625-635.

**Chen, S., Rouse, M.N., Zhang, W., Zhang, X., Guo, Y., Briggs, J. and Dubcovsky, J.** (2020) Wheat gene *Sr60* encodes a protein with two putative kinase domains that confers resistance to stem rust. *New Phytol*, **225**, 948-95

**Rouse M, Jin Y. 2011.** Stem rust resistance in A-genome diploid relatives of wheat. *Plant Dis* **95**: 941-944.

**Saintenac, C., Zhang, W., Salcedo, A., Rousse, M., Trick, H., Akhunov, E. and Dubcovsky, J.** (2013) Identification of wheat gene *Sr35* that confers resistance to Ug99 stem rust race group. *Science*, **341**, 783-786.

**Steuernagel, B., Periyannan, S.K., Hernandez-Pinzon, I., Witek, K., Rouse, M.N., Yu, G., Hatta, A., Ayliffe, M., Bariana, H., Jones, J.D., Lagudah, E.S. and Wulff, B.B.** (2016) Rapid cloning of disease-resistance genes in plants using mutagenesis and sequence capture. *Nat Biotechnol*, **34**, 652-655.

**Chen, S., Zhang, W., Bolus, S., Rouse, M.N. and Dubcovsky, J.** (2018b) Identification and characterization of wheat stem rust resistance gene *Sr21* effective against the Ug99 race group at high temperature. *PLoS Genet*, **14**, e1007287.
